# Rhizosphere microbial communities associated to rose replant disease: links to plant growth and root metabolites

**DOI:** 10.1038/s41438-020-00365-2

**Published:** 2020-09-01

**Authors:** B. Yim, A. Baumann, G. Grunewaldt-Stöcker, B. Liu, L. Beerhues, S. Zühlke, M. Sapp, J. Nesme, S. J. Sørensen, K. Smalla, T. Winkelmann

**Affiliations:** 1 Institute of Horticultural Production Systems, Leibniz Universität Hannover, 30419 Hannover, Germany; 2 Julius Kühn-Institut (JKI), Institute for Epidemiology and Pathogen Diagnostics, 38104 Braunschweig, Germany; 3 Institute of Pharmaceutical Biology, Technische Universität Braunschweig, 38106 Braunschweig, Germany; 4 Faculty of Chemistry and Chemical Biology (CCB), Technische Universität Dortmund, 44227 Dortmund, Germany; 5 Cluster of Excellence on Plant Sciences (CEPLAS), Institute for Population Genetics, Heinrich Heine University, 40225 Düsseldorf, Germany; 6 Section of Microbiology, Department of Biology, University of Copenhagen, 2100 Copenhagen, Denmark

**Keywords:** Plant physiology, Biotic

## Abstract

Growth depression of *Rosa* plants at sites previously used to cultivate the same or closely related species is a typical symptom of rose replant disease (RRD). Currently, limited information is available on the causes and the etiology of RRD compared to apple replant disease (ARD). Thus, this study aimed at analyzing growth characteristics, root morphology, and root metabolites, as well as microbial communities in the rhizosphere of the susceptible rootstock *Rosa* *corymbifera* ‘Laxa’ grown in RRD-affected soil from two sites (Heidgraben and Sangerhausen), either untreated or disinfected by γ-irradiation. In a greenhouse bioassay, plants developed significantly more biomass in the γ-irradiated than in the untreated soils of both sites. Several plant metabolites detected in *R. corymbifera* ‘Laxa’ roots were site- and treatment-dependent. Although aloesin was recorded in significantly higher concentrations in untreated than in γ-irradiated soils from Heidgraben, the concentrations of phenylalanine were significantly lower in roots from untreated soil of both sites. Rhizosphere microbial communities of 8-week-old plants were studied by sequencing of 16S rRNA, ITS, and cox gene fragments amplified from total community DNA. Supported by microscopic observations, sequences affiliated to the bacterial genus *Streptomyces* and the fungal genus *Nectria* were identified as potential causal agents of RRD in the soils investigated. The relative abundance of oomycetes belonging to the genus *Pythiogeton* showed a negative correlation to the growth of the plants. Overall, the RRD symptoms, the effects of soil treatments on the composition of the rhizosphere microbial community revealed striking similarities to findings related to ARD.

## Introduction

The severe growth depression of plants grown repeatedly at the same site is a typical symptom of replant disease (RD), which is predominantly observed in the family Rosaceae (e.g., apple, cherry, and rose). RD is already observed after a few replanting generations^1^. Symptoms on RD-affected apple plants include short internodes, smaller leaf area, significantly reduced aboveground growth and biomass, reduced and necrotic root growth, delayed flowering, and significantly declined yield compared to healthy plants^1–5^.

Extensive studies have been reviewed for apple RD (ARD) worldwide^3,6,7^. ARD has recently been defined as “harmfully disturbed physiological and morphological reaction of apple plants to soils that faced alterations in their (micro-)biome due to previous apple cultures^7^.” In contrast, very limited information is available for rose RD (RRD)^8^. However, cross-reactions are known. Yim et al.^9^ reported that the ARD-susceptible apple rootstocks M106 and M26 were significantly reduced in their shoot lengths (SLs) and biomasses when planted at sites previously cultivated with rose rootstocks compared to those grown on fumigated plots.

ARD symptoms at the microscopic level include a severely damaged root structure, root necrosis, blackening in the intercellular space of cortex tissue along with black inclusions in the cytoplasm—possibly filled with phenolic compounds—and decreased root hair formation^2,5^. A high content of total phenolic compounds in ARD-infected roots was reported previously^4,10^. Moreover, Weiß et al.^11^ detected the accumulation of a special group of phenolic compounds, i.e., biphenyls and dibenzofurans, in roots of M26 grown in ARD soil. This finding was supported by a strong upregulation of genes involved in the biosynthesis of these plant defense compounds^11,12^. Biphenyls and dibenzofurans are the typical phytoalexins of the rosaceous subtribe Malinae (formerly defined as subfamily Maloideae)^13^. Other phenolic compounds such as catechins and gallocatechins are produced as defense compounds by some members of the Rosaceae^14^. Analyses of secondary metabolites so far carried out in *Rosa* sp. were related to the pharmaceutical use of roses^15,16^. However, only little information is available about phytoalexins in roses.

The severity of ARD symptoms is dependent on the susceptibility of the plant species or genotype, as well as on various soil properties^1,5,6,9^. Site-dependent effects of ARD were reported to link to the indigenous soil microbiome of a given site, which was shaped by cropping histories and management practices^17,18^. Comparative analyses of the composition of the microbial communities in ARD and disinfected ARD soils were carried out at the DNA level using various methods, namely denaturing gradient gel electrophoresis fingerprinting^5,9^, amplicon sequencing of 16S rRNA gene fragments (for bacteria) and internal transcribed spacer (ITS) regions (for fungi)^4,17–20^, and shotgun metagenomics^21^. These studies recorded changes in the soil microbiome, possibly indicating a lack of plant growth-promoting microbes in ARD soils.

Based on culture-dependent approaches, several organisms associated to ARD were documented, e.g., actinomycetes^22^, *Pythium* sp.^23^, *Cylindrocarpon* sp., *Phytophthora* sp., *Rhizoctonia solani*^24–26^, and the endoparasitic nematode *Pratylenchus penetrans*^27^. In addition, recent studies confirmed several fungal endophytes isolated from ARD-infected apple roots (M26) to cause detrimental effects on the plants in inoculation experiments^28^. The symptoms induced by these isolated endophytes (*Ilyonectria crassa*, *Ilyonectria robusta*, *Calonectria* sp., *Dactylonectria torresensis*, *Leptosphaeria* sp., *Cadophora luteo-olivacea*, and *Cylindrocladiella* sp.) were chlorosis, necrosis to death of shoots, and browning of roots^28^. Root microscopic analyses revealed a strong colonization of ARD-affected roots by members of Actinobacteria and Nectriaceae (*Cylindrocladiella*, *Ilyonectria*, *Calonectria*, and *Dactylonectria*)^2,28^.

Obviously, ARD is caused by a disease complex and it is a case of a negative soil-plant feedback. However, up to date the etiology of ARD is not fully understood. Even less is known about RRD. Actinobacteria and *P. penetrans* were proposed to be involved^8,29^. However, detailed scientific investigations of the soil, rhizosphere, and root microbiome under RRD conditions have not yet been carried out. Nevertheless, RRD is affecting rose rootstock production and field production of garden roses in tree nurseries, resulting in severe economic losses. Changing sites of production is often not feasible due to specialization and a lack of virgin soil. Soil disinfection is not environmentally friendly and cost intensive. Thus, efforts are needed to better understand the etiology of RRD and to develop measures to overcome the problem. Motivated by the recently achieved insights into ARD, this study aimed at a concise characterization of rose rootstock responses to RRD soils by investigating the rhizosphere microbiome (bacteria, fungi, and oomycetes), the root morphology and phenolic secondary metabolite profiles.

The analyses were conducted using the RRD-susceptible rootstock *Rosa corymbifera* ‘Laxa’ grown in untreated and γ-irradiated RRD soils from two sites under greenhouse conditions. The following hypotheses were addressed in the present study. (1) The composition of the rhizosphere microbiome (bacteria, fungi and oomycetes) is significantly affected by the soil treatments. (2) The enhanced growth of the *R. corymbifera* ‘Laxa’ plants is linked to a reduction in the relative abundance of taxa known as potential plant pathogens. (3) Symptoms on RRD-affected roots are similar to those observed for ARD-affected apple roots. (4) The secondary metabolite profiles in RRD-affected roots differ from those detected in roots from disinfected RRD soil.

## Results

### Growth of *R. corymbifera* ‘Laxa’ in RRD-affected soils

**Fig. 1:**
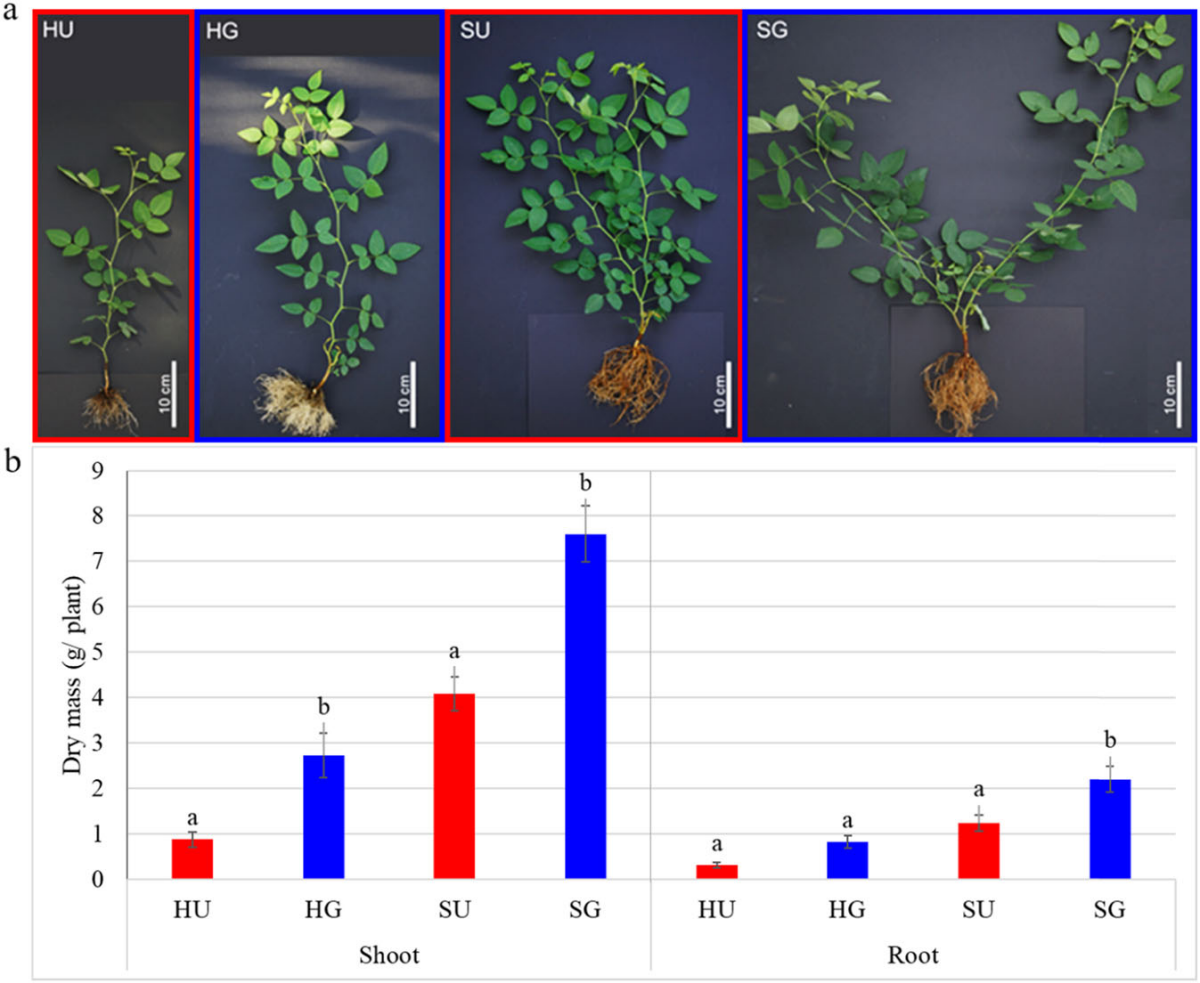
Effects of RRD on *R. corymbifera* ‘Laxa’. Appearance (**a**) and biomass (**b**) at the end of the bioassay, i.e., after 8 weeks of growth in soil from Heidgraben (H) and Sangerhausen (S), either untreated (U) or γ-irradiated (G). Data are means ± SD (*n* = 10 and 5 for shoot and root dry masses, respectively). Letters indicate significant differences between variants within sites (*t*-test, *p* < 0.05)

To prove the RD severity of the two soils from Heidgraben (H) and Sangerhausen (S), plant growth in the untreated soils (HU and SU) was compared to that in γ-irradiated soils (HG and SG), respectively. Already after two weeks of planting, significantly higher growth of the *R. corymbifera* ‘Laxa’ plants in terms of the main shoot length (SL) was observed in the G soil variants of both sites compared to that in the U soils (Supplementary Fig. S2a, b). In soil H, the difference increased over time, whereas in soil S the main shoots did differ significantly in length in weeks two to six, but no longer after seven weeks. This was due to pronounced branching in soil S (Fig. 1). When dry masses were compared, clearly reduced shoot dry mass (SDM) was recorded for plants grown in the U soil variants HU and SU (Fig. 1). The root dry mass (RDM) of plants in soil H was only slightly reduced in the U compared to the G variant, whereas in soil S the difference was significant (Fig. 1). Overall, *R. corymbifera* ‘Laxa’ plants grew significantly better in the loamy soil from site S.

### Microscopic symptoms on *R. corymbifera* ‘Laxa’ roots affected by RRD

**Fig. 2:**
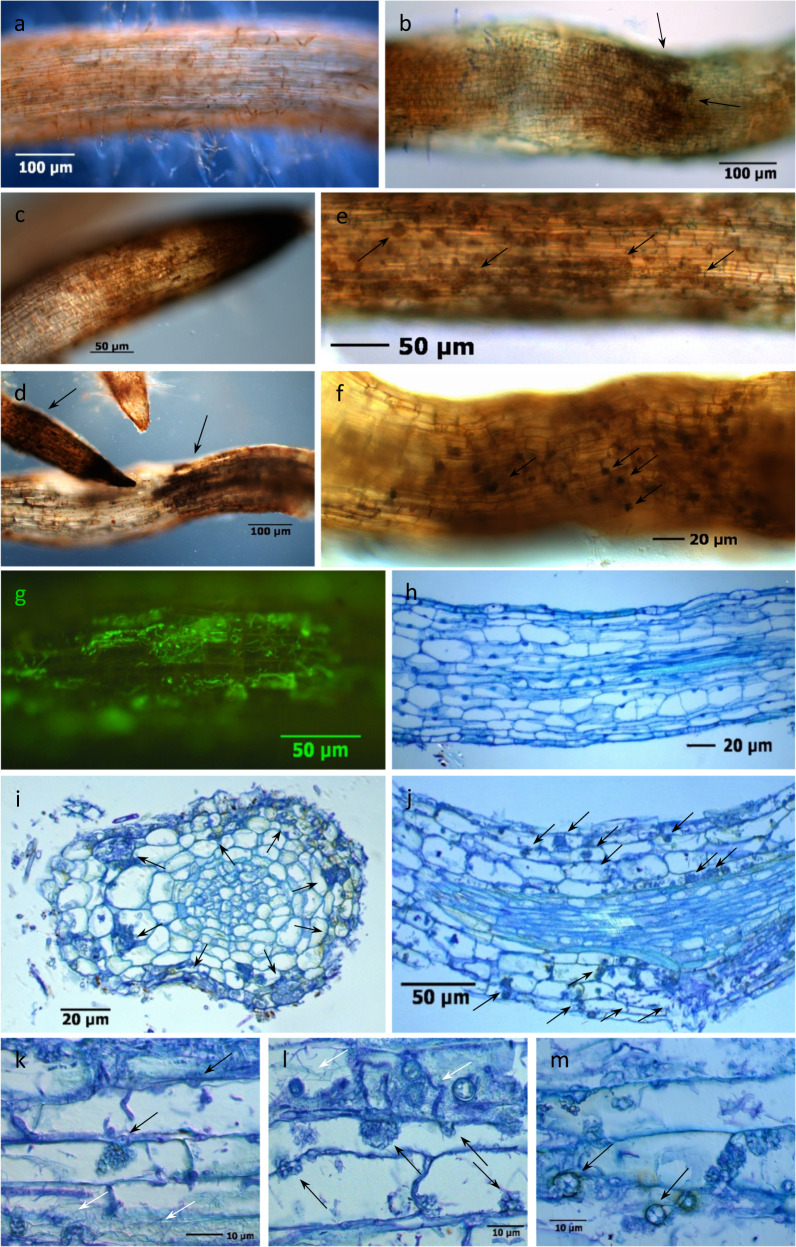
Symptoms of replant disease in fine roots of *R. corymbifera* ‘Laxa’. Symptoms were detected after eight weeks of cultivation in RRD soil from the site Heidgraben (H) in whole-mount samples (**a**–**g**) and thin sections stained with toluidine blue (**f**–**m**). **a** Healthy fine root with intact root hair zone; **b** constricted root structure and brown necrotic zones with black cell clusters (arrows) in a toluidine blue-stained root segment; **c**, **d** black root tips and clusters (arrows) of necrotic rhizodermal and cortical cells; **e** fine root with intracellular cauliflower-like (CF) fungal structures (arrows) in necrotic rhizodermal and cortex cells; **f** CF structures and black cell inclusions (arrows); **g** green fluorescent Actinobacteria in cortex cells after FUN^®^1 cell vital staining. **h** Longitudinal thin section through healthy fine root tissues with rhizodermis R, cortex C, endodermis E, stele St, and xylem X; **i** cross section of an infected fine root with fungal hyphae and CF structures (arrows) in necrotic cortex cells; **j** infected fine root with fungal CF structures and hyphae (arrows) in rhizodermal and cortical cells; **k** thick fungal hyphae, intercellular entry point and developing intracellular CF structure (black arrows) and Actinobacteria (white arrows); **l** mixed infection with a CF structure-forming fungus (black arrows), rod-shaped bacteria, and thread-like Actinobacteria (white arrows); **m** cortex cells with fungal CF structures and round-shaped fungal chlamydospores (arrows)

To reveal typical symptoms on roots grown in RRD soils, fine roots of *R. corymbifera* ‘Laxa’ were investigated using microscopy. In both whole-mount samples and thin sections of diseased fine roots that were grown in HU and SU soils, damage and infections were visible when comparing them to healthy roots (Fig. 2a). In fresh samples, symptoms of RRD were root constrictions, root tip necrosis and dying back, clustered blackening of cortex cells, and black cytoplasmic inclusion bodies in rhizodermal cells (Fig. 2b–f). The association of fungal infections with some of these tissue alterations was obvious. Intracellular condensed nodular fungal structures in the form of a cauliflower head (here named CF structures) and chlamydospores, which were strongly attributed to Nectriaceae, appeared after fungal penetration into the cortex tissue layers of fine roots (Fig. 2j-m). Actinobacteria colonized the root surface and the rhizodermal and cortical cell layers, often in combination with fungal infections. Their filamentous structures could be easily distinguished from coccoid or rod-shaped bacteria by fluorescence microscopy and in stained tissue thin sections (Fig. 2g, k, l).

### Secondary metabolites of *R. corymbifera* ‘Laxa’ affected by RRD soil

**Fig. 3:**
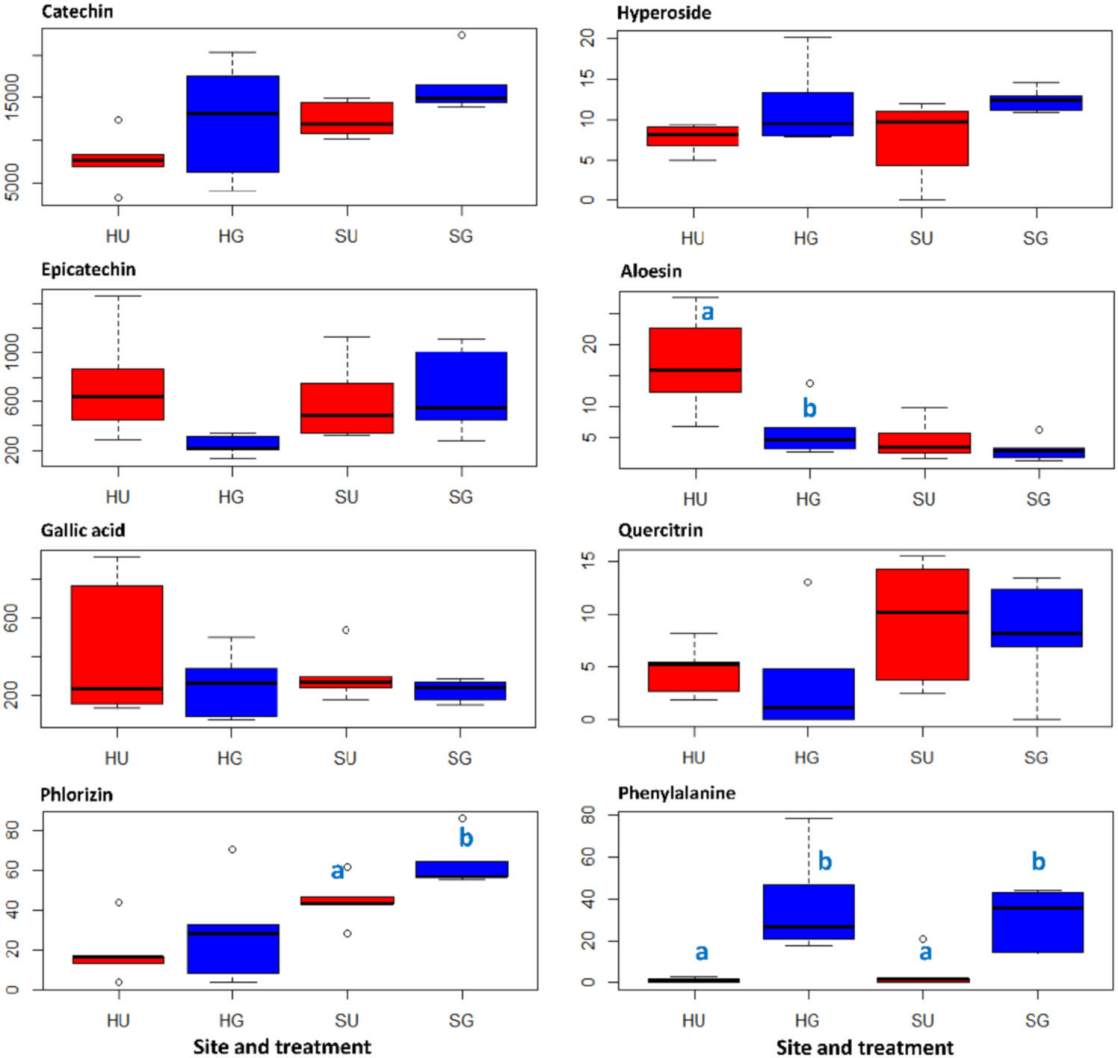
Concentration (µg g^−1^ DM) profiles of selected metabolites in roots of *R. corymbifera* ‘Laxa’. Contents of the secondary metabolites (catechin, epicatechin, gallic acid, phlorizin, hyperoside, aloesin, and quercitrin) and a primary metabolite (phenylalanine) were detected by HPLC-HR-MS and GC-MS (epicatechin and gallic acid). HU and SU, untreated RRD soils from Heidgraben and Sangerhausen, respectively; HG and SG, γ-irradiated RRD soils from Heidgraben and Sangerhausen, respectively. Letters indicate significant differences in the contents of compounds between untreated and γ-irradiated samples within a site, *t*-test at *p* < 0.05 and *n* = 5

Roots of *R. corymbifera* ‘Laxa’ harvested eight weeks after planting were used to analyze the secondary metabolite content. In total, seven secondary metabolites were identified by high performance liquid chromatography coupled to high resolution mass spectrometry (HPLC-HR-MS) and gas chromatography coupled to mass spectrometry (GC-MS) (Fig. 3). Furthermore, one primary metabolite that exhibited a treatment-dependent pattern was detected. The contents of catechin, epicatechin, gallic acid, hyperoside, and quercitrin did not show statistically significant differences between roots from U and G soils at both sites. However, the aloesin content was significantly increased in roots grown in HU soil compared to that found in roots from HG soil. In contrast, the phlorizin content was significantly decreased in roots grown in SU soil compared to that detected in roots from SG soil. The content of the primary metabolite phenylalanine was significantly lower in roots grown in U soil compared to that found in roots from G soil, which was true for both sites (Fig. 3). No new natural compound that was associated to RRD was found via HPLC-HR-MS and GC-MS screenings at both sites (H and S).

### Microbial communities in the *R. corymbifera* ‘Laxa’ rhizosphere affected by soil variant

#### Bacterial communities

Except for a significant reduction in operational taxonomic unit (OTU) numbers in SG compared to SU soil, bacterial α-diversity indices were not significantly altered due to treatment effects at both sites (Supplementary Table S3).

**Fig. 4:**
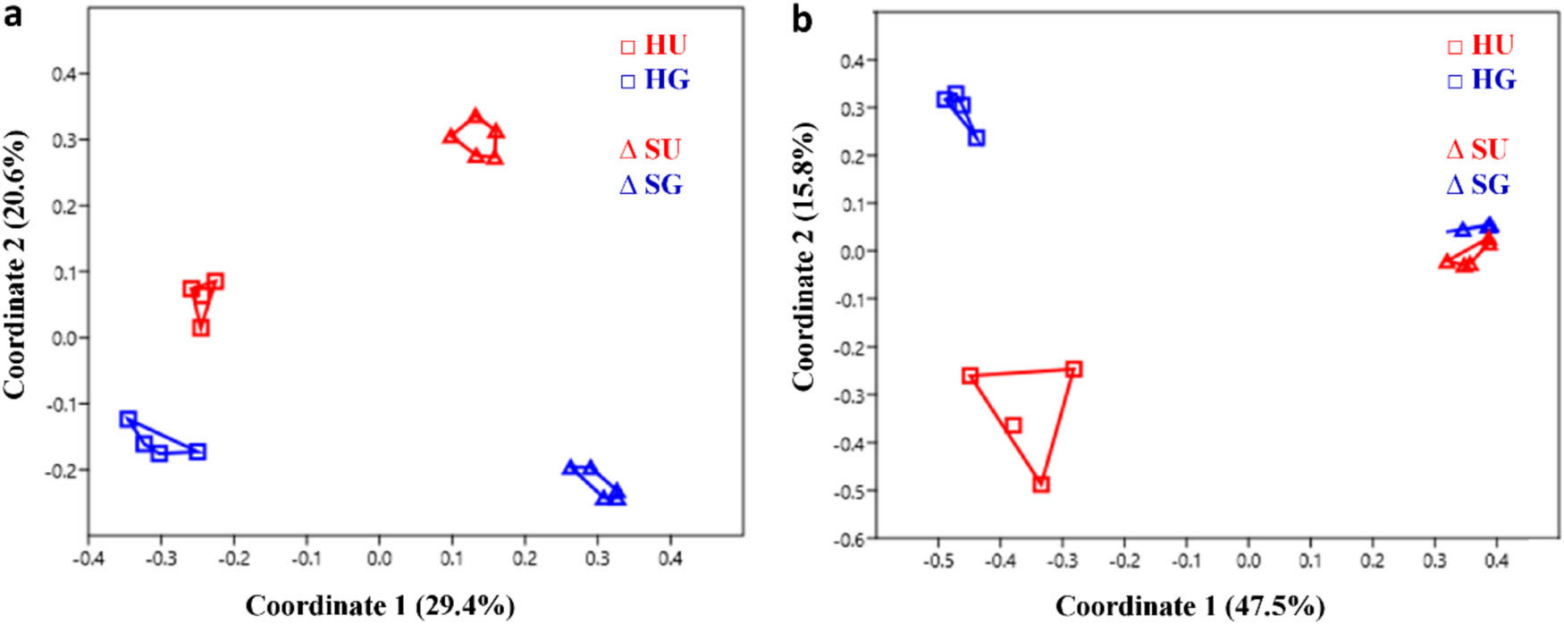
Microbial community compositions in the rhizosphere of *R. corymbifera* ‘Laxa’ grown for eight weeks in untreated (U) and γ-irradiated (G) RRD soils from two sites (Sangerhausen S and Heidgraben H). Bacterial (**a**) and fungal (**b**) community compositions were revealed by principal coordinate analysis (PCoA) using Bray–Curtis distance metric, based on operational taxonomic units (OTUs). Past3, *n* = 4 and 5 for sites H and S, respectively, for **a** and *n* = 4 for **b**, except for SU with *n* = 5

The principal coordinate analysis (PCoA) revealed a clear clustering of the bacterial community compositions in the rhizosphere soil from both sites (H and S) (Fig. 4a). The significantly distinct bacterial community composition between the two sites was confirmed by the Anosim test (*R*-value = 0.9, *p*-value = 0.0001). The rhizosphere bacterial community composition was significantly affected by γ-irradiation at both sites, with a higher effect at site S (*R*-value = 1, *p*-value = 0.01) compared to site H (*R*-value = 0.8, *p*-value = 0.03) (Fig. 4a).

**Fig. 5:**
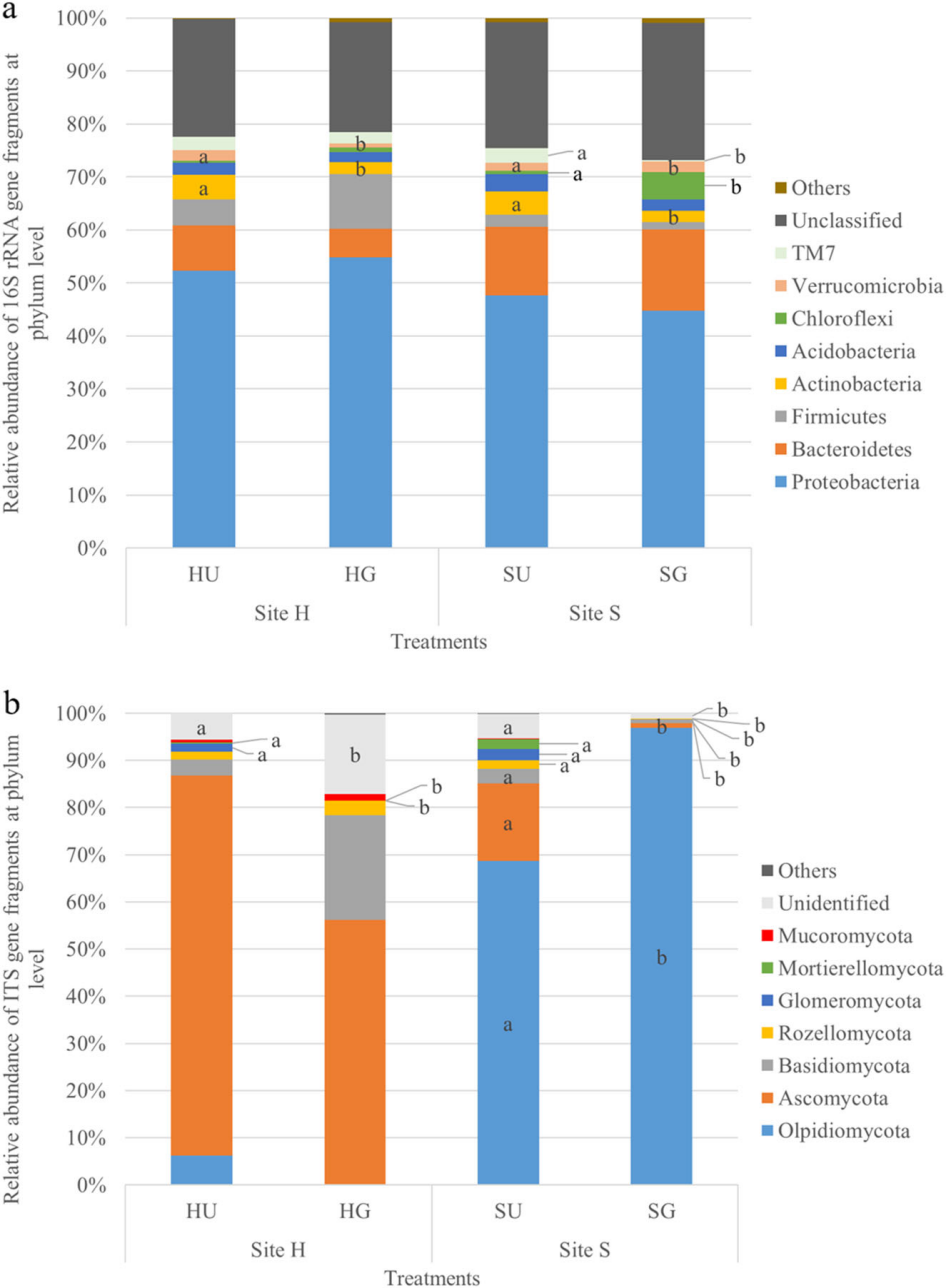
Dominant microbial phyla in the rhizosphere of *R. corymbifera* ‘Laxa’ grown for eight weeks in untreated (U) and γ-irradiated (G) RRD soils from two sites (H and S). The relative abundance of dominant bacterial (**a**) and fungal (**b**) phyla (>1%) is indicated. Letters indicate significant differences between treatments within a site and phylum, *t*-test, *p* < 0.05 and *n* = 4 and 5 for sites H (Heidgraben) and S (Sangerhausen), respectively (**a**), *n* = 4, except for SU with *n* = 5 (**b**)

On the phylum level, sequences belonging to 17 bacterial phyla were identified, 8 of which were considered rare because of relative abundances below 1% (data not shown). The most dominant relative abundance was recorded for the bacterial phylum Proteobacteria (both sites), i.e., sharing up to 54.8% in HG soil (Fig. 5a). Only members of Actinobacteria were commonly affected by the treatment, being significantly reduced in relative abundance in SG and HG soils.

**Fig. 6:**
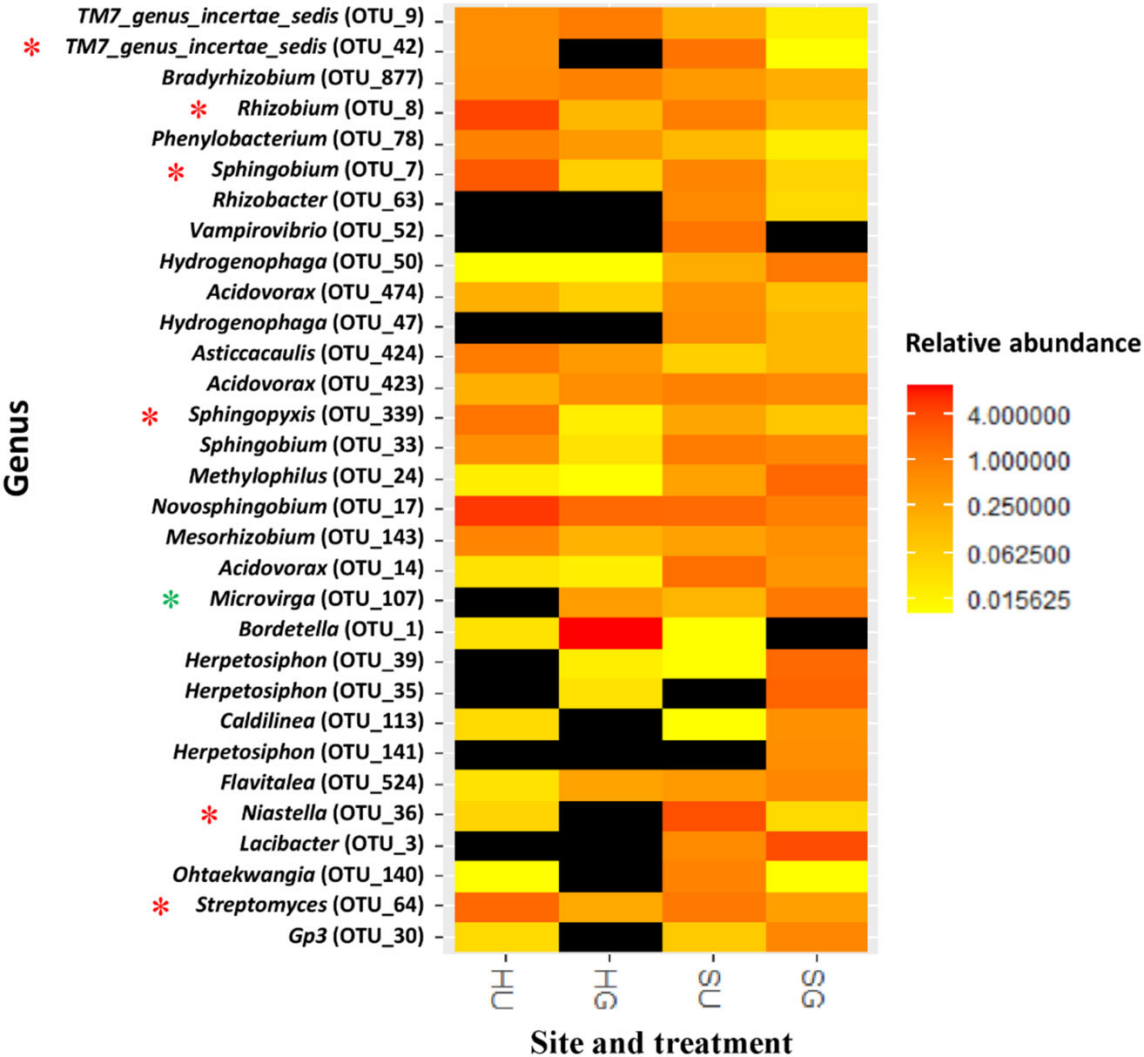
Effects of soil treatments on relative abundance of bacteria at the genus level (>0.5%) in the rhizosphere of *R. corymbifera* ‘Laxa’. Plants were grown for eight weeks in untreated (U) and γ-irradiated (G) RRD soils from two sites (S and H). Asterisks (*) in red and green color indicate significantly decreased and increased relative abundances of bacteria, respectively, in γ-irradiated compared to untreated RRD soils at both sites (H and S). *T*-test at *p* < 0.05, *n* = 4 and 5 for sites H (Heidgraben) and S (Sangerhausen), respectively. OTU, operational taxonomic unit. The relative abundance of the respective taxon below detection limit is indicated in black color

On the genus level, several shifts in bacterial relative abundances due to soil treatments were mostly site-specific (Fig. 6 and Supplementary Table S4). However, common changes in the relative abundance of several bacterial taxa were observed in soil from both sites, which might point to general RRD-related effects. Among them, the bacterial genera *Streptomyces*, *Niastella*, *Sphingobium*, *Sphingopyxis*, *Rhizobium*, and *TM7_genus_incertae_sedis* were significantly reduced in their relative abundances in HG and SG soils. In contrast, only one bacterial genus, *Microvirga*, was found in significantly increased relative abundance in HG and SG soils (Fig. 6 and Supplementary Table S4). Interestingly, three of these common responders (*Streptomyces*, *Sphingobium*, and *Rhizobium*) showed a significant negative correlation with shoot and root biomass of the *R. corymbifera* ‘Laxa’ plants, from which the rhizosphere samples were taken (Table 1).

**Table 1:** Pearson’s correlation between relative abundances (>0.5%) of 16S rRNA gene fragments detected in the rhizosphere and shoot dry mass (SDM) and root fresh mass (RFM) of *R. corymbifera* ‘Laxa’ eight weeks after planting

Genus	OTU_ID	Relative abundance (mean ± SD, *n* = 18)	SDM		RFM	
			*r*-Value	*p*-Value	*r*-Value	*p*-Value
*Bradyrhizobium*	OTU_877	0.50 ± 0.36	−0.63	0.00	−0.20	0.42
*Herpetosiphon*	OTU_35	0.59 ± 1.12	0.62	0.01	0.30	0.23
*Lacibacter*	OTU_3	1.21 ± 1.80	0.84	0.00	0.59	0.01
*Methylophilus*	OTU_24	0.61 ± 0.83	0.89	0.00	0.54	0.02
*Novosphingobium*	OTU_17	2.40 ± 2.01	−0.72	0.00	−0.83	0.00
*Rhizobium*	OTU_8	1.32 ± 1.83	−0.68	0.00	−0.81	0.00
*Sphingobium*	OTU_7	0.86 ± 1.73	−0.46	0.05	−0.58	0.01
*Streptomyces*	OTU_64	0.89 ± 0.85	−0.55	0.02	−0.62	0.01

*OTU* operational taxonomic unit

Data present only those taxa that showed significant correlation to at least one growth parameters

#### Fungal communities

As described above for bacteria, the G treatment also significantly reduced the OTU numbers and species richness (Chao1) at site S for fungal ITS sequences (Supplementary Table S5). As observed before for bacterial communities, the Anosim test (*R*-value = 1, *p*-value = 0.0001) indicated significant differences in rhizosphere fungal community compositions between the two sites. In contrast to our observations on bacteria, the fungal community composition was significantly and stronger affected by the G treatment at site H (*R*-value = 0.8, *p*-value = 0.03) than at site S (*R-*value = 0.3, *p*-value = 0.04) (Fig. 4b).

On the phylum level, it became obvious that both soils differed strongly with Ascomycota and Olpidiomycota, being the phyla of highest relative abundance in soil from sites H and S, respectively (Fig. 5b). A common response to the G treatment was noted for Glomeromycota and Mortierellomycota with a significant reduction in relative abundances compared to those in U soil (Fig. 5b).

Considering the fungal genera with significant differences in relative abundances between U and G soils, OTUs belonging to *Nectria* were commonly and significantly decreased in the rhizosphere at sites H and S (Table 2). In addition, the relative abundance of this fungal genus indicated a significant negative correlation to the biomass of *R. corymbifera* ‘Laxa’ (Table 3). As members of *Nectria* contain a complex of several closely related species that are difficult to separate taxonomically, a BLAST search against the NCBI database (nucleotide collection, nr/nt) was applied and the OTUs belonging to the fungal genus *Nectria* could be annotated to *Neonectria* sp. and *Cylindrocarpon* sp.

**Table 2:** Relative abundances (>0.5%) of fungi at genus levels detected in the rhizosphere of *R. corymbifera* ‘Laxa’ grown for eight weeks in untreated (U) and γ-irradiated (G) RRD soil from two sites (S and H)

Phylum	Genus	Pipeline BLAST	NCBI BLAST	OTU_ID	Site H		Site S	
		Species			HU	HG	SU	SG
Ascomycota	*Pilidium*	*Pilidium concavum* SH182030.07FU		OTU_1773	21.01 ± 42.01	0.00 ± 0.00	0.04 ± 0.03 a	0.00 *±* 0.01 b
	*Nectria*^*a*^		*Neonectria* sp. TB101 (*E*-value, 6e − 35, Ident, 81%)	OTU_1869	15.02 ± 11.48 a	0.01 ± 0.01 b	0.16 ± 0.11 a	0.00 *±* 0.00 b
	*Nectria*		*Cylindrocarpon* sp. W5-1 (*E*-value, 3e − 52, Ident, 87%)	OTU_2381	2.44 ± 2.16	0.00 ± 0.00	0.14 ± 0.11 a	0.00 ± 0.00 b
	*Archaeorhizomyces*			OTU_5391	0.03 ± 0.05 a	0.81 ± 0.41 b	0.00 ± 0.00	0.00 ± 0.00
Olpidiomycota	*Olpidium*	*Olpidium brassicae* SH216672.07FU		OTU_32	0.13 ± 0.11	0.02 ± 0.03	67.93 ± 13.76	81.38 ± 19.85

*OTU* operational taxonomic unit

Mean ± SD. Letters indicate significant differences between treatments within site and genus, *t*-test, *p* < 0.05 and *n* = 4, except that SU, *n* = 5. Only significant OTUs affected by treatments are presented

^*a*^Common responder found at both sites

**Table 3:** Pearson’s correlation between relative abundances (>0.5%) of fungal ITS gene fragments detected in rhizosphere and shoot dry mass (SDM) and root fresh mass (RFM) of *R. corymbifera* ‘Laxa’ eight weeks after planting

Genus	OTU_ID	Relative abundance (mean ± SD, *n* = 17)	SDM		RFM	
			*r*-Value	*p*-Value	*r*-Value	*p*-Value
*Nectria*	OTU_1869	3.58 ± 8.22	−0.53	0.03	−0.65	0.00
*Nectria*	OTU_2381	0.61 ± 1.40	−0.50	0.04	−0.59	0.01
*Olpidium*	OTU_32	39.16 ± 39.86	0.71	0.00	0.41	0.10

*OTU* operational taxonomic unit

Data present only those taxa that showed significant correlation to at least one of the growth parameters

#### Oomycete communities

Low OTU numbers of 6–12 and no significant treatment effects on oomycete α-diversities were obtained using the *cox 2* sequence analyses (Supplementary Table S6).

Also, the oomycete community composition differed significantly between the two sites H and S, but differences were not as pronounced as those of the bacteria and fungi (PCoA and Anosim test with *R*-value = 0.3 and *p*-value = 0.01, Supplementary Fig. S3). At site H, the G treatment significantly altered the oomycete community composition (*R-*value = 0.8, *p*-value = 0.01) in the rhizosphere, whereas no significant effect of the treatment was recorded at site S (*R*-value = 0.20, *p*-value = 0.054) (Supplementary Fig. S3).

On the genus level, the highest relative abundance in U soil was detected for *Peronospora* at both sites, sharing up to 53.1 ± 33.4% in HU soil (Table 4) and being reduced in relative abundance after the G treatment. In the present study, the sequences belonging to oomycetes *Peronospora*, *Peronospora potentillae-anserinae*, were negatively correlated to plant growth (Supplementary Table S7). No common responders were found within the oomycete genera, which was at least partly due to the high variation between replicates. However, for soil from site S, members of *Globisporangium ultimum*, *G. attantheridium, Eraphthora butleri*, and *Pythiogeton ramosum* were significantly lower in their relative abundance in the G soil. On the other hand, only one species, *Myzocytiopsis subuliformis*, was detected in highly increased relative abundance in HG compared to HU soil (Table 4).

**Table 4:** Relative abundances (>0.5%) of oomycete genera/species detected in the rhizosphere of *R. corymbifera* ‘Laxa’ grown for eight weeks in untreated (U) and γ-irradiated (G) RRD soil from two sites (S and H)

Phylum	Genus	Species	OTU_ID	Site H		Site S	
				HU	HG	SU	SG
Oomycetes	*Globisporangium*	*Globisporangium mamillatum*	OTU_1	20.13 ± 31.71	7.12 ± 7.89	29.70 ± 19.64	60.81 ± 42.44
		*G. ultimum*	OTU_10	0.00 ± 0.00	0.00 ± 0.00	0.62 ± 0.38 a	0.00 ± 0.00 b
		*Globisporangium attrantheridium*	OTU_279	0.03 ± 0.06	0.05 ± 0.12	9.68 ± 8.27 a	0.58 ± 1.08 b
		*Globisporangium apiculatum*	OTU_53	0.00 ± 0.00	0.05 ± 0.12	0.23 ± 0.32	7.55 ± 14.50
		*Globisporangium heterothallicum*	OTU_9	0.00 ± 0.00	0.00 ± 0.00	6.50 ± 10.47	0.58 ± 1.16
	*Myzocytiopsis*	*M. subuliformis*	OTU_5	0.00 ± 0.00 a	53.65 ± 40.97 b	0.00 ± 0.00	0.74 ± 1.48
	*Peronospora*	*P. potentillae-anserinae*	OTU_2	53.10 ± 33.35 a	0.23 ± 0.28 b	23.97 ± 41.95	1.00 ± 1.75
		*E. butleri*	OTU_3	1.19 ± 1.23	20.80 ± 24.55	3.97 ± 3.02 a	0.26 ± 0.52 b
	*Pythiogeton*	*P. ramosum*	OTU_349	2.42 ± 3.17	0.15 ± 0.14	1.88 ± 1.56 a	0.00 ± 0.00 b
	*Pythium*	*Pythium acanthicum*	OTU_14	0.00 ± 0.00	0.00 ± 0.00	0.31 ± 0.69	0.74 ± 1.48
		*Pythium monospermum*	OTU_28	0.00 ± 0.00	0.00 ± 0.00	0.03 ± 0.06	0.61 ± 0.80
	*Phytophthora*	*Phytophthora andina*	OTU_11	0.71 ± 1.42	0.46 ± 1.04	7.33 ± 10.49	0.16 ± 0.24
		*Phytophthora cinnamomi*	OTU_34	0.00 ± 0.00	0.00 ± 0.00	1.11 ± 1.90	0.06 ± 0.13
		*Phytophthora bisheria*	OTU_7	12.94 ± 17.18	2.81 ± 6.29	0.03 ± 0.06	24.81 ± 49.01

*OTU* operational taxonomic unit

Mean ± SD. Letters indicate significant differences between treatments within site and genus, *t*-test, *p* < 0.05 and *n* = 4 for HU and SG and *n* = 5 for HG and SU

## Discussion

### Bioassay proved both soils to be affected by RRD

Differences in soil texture, pH, and plant available nutrients (Supplementary Tables S1 and S2) of the two RRD sites resulted in growth differences of the *R. corymbifera* ‘Laxa’ plants. Significantly higher biomass was observed in the loamy soil from site S (Fig. 1 and Supplementary Fig. S2). This finding was in agreement with previous studies of apple plants demonstrating a better growth of apple in loamy soils^1,5,17,18^. A significant reduction in SL and plant biomass in U soils compared to G soils from both sites (Fig. 1 and Supplementary Fig. S2) provided evidence that the greenhouse bioassay was suitable for the evaluation of RRD in a small soil volume (1 L pot) and within a short period of time (symptoms on plant growth were recorded earliest after two weeks). Furthermore, the significantly increased growth in the G soils compared to the respective U soils clearly indicated that both soils were affected by RRD. The stronger relative reduction in growth of *R. corymbifera* ‘Laxa’ plants in soil from site H suggests that RRD in this soil was more severe than in the soil from site S. This could be due to the higher sand content, corresponding to a bigger pore volume or to the fourth rose replanting cycle at this site.

### Microscopic RRD indicators in *R. corymbifera* ‘Laxa’ roots display striking similarities to ARD symptoms in apple roots

This study provided evidence that the early symptoms of RD in rose fine roots are similar to those in apple roots. Necrosis, often associated with the development of CF structures and black cytoplasmic inclusions, and blackening of rhizodermal and cortical cells, singly or in clusters, have been described in studies with different apple genotypes, which were cultivated in ARD-affected soils of three different sites^2^. These findings confirm the assumption of Szabo^30^, who suggested that similar pathogens are involved in RRD and ARD. Detailed analyses of various early disease parameters of RRD, including the significantly disturbed vitality of rhizodermal cells, have recently been reported^31^. In particular, the very early and frequently occurring CF-forming fungi in rose, which trigger necrotic plant reactions, point to their essential role in the origin of the disease. Meanwhile, the identities of many isolates of the CF-forming fungi in rose and in apple plants have been shown to belong to different genera of the Nectriaceae, which can simultaneously infect one plant^31,32^. The ability of these highly infectious Nectriaceae to form numerous chlamydospores, which could serve as inoculum reservoir in soils, underlines their pathogenic potential. Nectriaceous fungi (formerly classified as *Cylindrocarpon*-like fungi) have been repeatedly mentioned as causal actors in the ARD complex^3,19,24,26,33^. It has recently been confirmed that Nectriaceae isolates also contribute to the disease in replant-affected rose roots, with more severe symptoms in roots from HU than SU soils^31^. This result is supported by higher relative abundance of Ascomycota (Fig. 5b), and especially of *Nectria*, OTUs (Table 2) in the untreated RRD soils.

Actinobacteria have frequently been studied in bacterial communities associated with ARD and have been considered both as positive for plant growth^4,19^ and as causal agents of ARD^22,29^. In our RRD studies, the visible frequency of Actinobacteria was significantly higher in HU and SU than in the respective G treatments^31^, which complements the 16S rRNA amplicon sequencing data (Fig. 5a) and the data for *Streptomyces* (Supplementary Table S4 and Fig. 6). However, the role of the Actinobacteria remains unsolved, as they might invade damaged tissue as primary colonizers or as successors of pathogenic fungi. The wide spectrum of beneficial and harmful Actinobacteria calls for further detailed studies on the species level.

### Secondary metabolites in RD-affected *R. corymbifera* ‘Laxa’ roots

Apple roots grown in ARD soils accumulate significantly higher amounts of biphenyl and dibenzofuran phytoalexins compared to roots grown in γ-irradiated ARD soils^11,12,34^. This led us to look for a comparable upregulation of phenolic secondary metabolites in rose roots. Catechin, epicatechin, and gallic acid were detected in high concentrations; however, neither consistently nor significantly affected by the soil variant (Fig. 3). Likewise, the data confirm a previous study that reported the presence of catechin, epicatechin, and various other prominent polyphenols (e.g., phlorizin and phloretin) in rose roots and root tips^35^. Gallic acid, commonly acting as an antioxidant, was detected in rose hips^15^. Phlorizin showed a clear trend of higher concentrations in roots grown in G soils (Fig. 3). Phlorizin has been proposed to be a phytoanticipin^36^. It may be assumed that the microbes in U soils induced the hydrolysis of phlorizin to its aglycone phloretin, which has a much higher antimicrobial activity^37^, and might be exuded into the soil. Six of the detected compounds are components of the phenylpropanoid and flavonoid pathways, as illustrated in Supplementary Fig. S4. Interestingly, phenylalanine also showed a clear trend of higher concentrations in roots grown in G soils. Phenylalanine yields cinnamic acid, which is the starting compound of the phenylpropanoid pathway. In U soils, the massive damage of roots observed microscopically is likely to be associated with an increased deposition of lignin, which is a product of phenylpropanoid metabolism. Thus, the increased carbon flow from phenylalanine to phenylpropanoids leads to a decreased level of this amino acid. The remarkably high variation in the metabolite contents between single plants might be due to either fast dynamic responses or different responses in different parts of the root system, which is also reflected by the patchy appearance of RRD.

Interestingly, the aloesin content was significantly higher in roots grown in HU soil than in roots grown in HG soil (Fig. 3). Aloesin was reported to be a typical secondary metabolite of the plant genera *Rheum* and *Aloe*, and exhibited both antimicrobial and free radical scavenging activities^38^. Therefore, aloesin might function as a phytoalexin in rose. Similar to flavonoids, it is a derivative of polyketide metabolism. To the best of our knowledge, it is the first time that aloesin was detected in rose.

### Soil- and treatment-dependent shifts in the rhizosphere microbiome

Overall, site- and treatment-dependent effects resulted in separate clustering of the rhizosphere bacteria, fungi, and oomycetes (PCoA; Fig. 4 and Supplementary Fig. S3). Significant differences (Anosim test) in the rhizosphere microbiome composition between the two RRD sites (H and S) and the treatments (HU vs. HG, SU vs. SG) were in agreement with previous findings at different ARD sites and treatments^5,17,18^. Site-dependent effects on the soil microbiome pointed to the fact that the indigenous soil microbiome is shaped by chemical and physical properties of the soil, cultivation histories, and soil management practices^39,40^.

γ-Irradiation of the RRD soil aimed at an elimination of the deleterious soil biota causing RRD and at altering soil microbial community composition and diversity, subsequently leading to favorable conditions for plant growth. The G treatment targets all sensitive organisms (neutral, beneficial, and pathogenic microbes), but the effects depend on the doses of γ-rays. In the current investigation, sequences affiliated to the bacterial phylum Actinobacteria were significantly reduced in relative abundance in G compared to U soils (at both sites, Fig. 5a). This finding well corresponds to the microscopic observation of Actinobacteria in RRD symptomatic roots (Fig. 2). It is also in line with data obtained in ARD studies^17^ but contradicts the data of a recent metagenome study, in which slightly higher read numbers for *Streptomyces* were found in control (grassland) than in ARD soil^21^. OTUs of the genus *Streptomyces* were detected in significantly lower relative abundance in both G variants and were negatively correlated to shoot and root growth of *R. corymbifera* ‘Laxa’ (Table 1). In contrast, Nicola et al.^4^ observed members of *Streptomyces* to be enhanced in ARD soil treated with the soil fumigant dazomet and to be positively correlated to the shoot growth of apple trees of the cultivar ‘Fuji Fubrax’ grafted on rootstock M9. This clearly shows the limitation of 16S amplicon sequencing, primarily short sequence lengths, by which OTUs can only be assigned to the genus level^41^. However, the genus *Streptomyces* comprises plant pathogenic species like those causing common scab on potato^42^, whereas other *Streptomyces* are used as biocontrol agents due to their antimicrobial metabolites and as biofertilizers due to their plant growth-promoting effects^43^. In the context of ARD, *Streptomyces* strains were found increased after *Brassica juncea* seed meal biofumigation and were regarded as pathogen suppressive^44^.

A negative correlation of *Novosphingobium*, *Rhizobium*, and *Sphingobium* members to the biomass of the *R.* *corymbifera* ‘Laxa’ plants (Table 1) was also observed by Franke-Whittle et al.^19^ who investigated *Malus* rootstock M9 rhizosphere bacteria associated with ARD. The significant reduction in the relative abundances of *Novosphingobium*, *Sphingobium*, and *Sphingopyxis* in G soil concurred with better plant growth in our study. However, members of these genera are known as beneficial rhizosphere bacteria^45^. The high relative abundance detected in the U soil (both sites) might be related to their role in degradation of phenolic compounds, which either remained in the soil as residues from previous plantings or are exuded from the current cultures^46^. Several phenolic compounds were also detected in roots (Fig. 3) and root exudates (data not shown) of *R. corymbifera* ‘Laxa’ in the present study. Furthermore, several genes involved in phenolic compound degradation in apple rhizosphere samples from ARD and control soils were also recently reported^21^.

A positive correlation of sequences affiliated to *Lacibacter*, *Methylophilus* and *Herpetosiphon* to SDM or RDM of *R. corymbifera* ‘Laxa’ plants was observed, and they were enhanced in relative abundances by the G treatment in S soil (Table 1, Fig. 6, and Supplementary Table S4). The proliferation of these bacteria was likely due to niche competition for the recolonization after soil treatments within the eight weeks of cultivation in the greenhouse. This assumption was confirmed by a correlation analysis showing that the relative abundances of *Lacibacter*, *Methylophilus* and *Herpetosiphon* were negatively correlated to those of several other bacterial genera (*Novosphingobium*, *Bradyrhizobium*, and *Streptomyces*), the fungal genus *Nectria*, and the oomycetes genus *Peronospora* (Supplementary Fig. S5).

Pronounced site-dependent effects on dominant fungal taxa were observed in the present study, i.e., for members belonging to Olpidiomycota at site S and Ascomycota at site H (Fig. 5b). The relative abundance of the fungal genus *Olpidium* was higher in SU than in HU soils, which was in contrast to the detected relative abundance of *Nectria* (Table 2). This could also be due to antagonistic activities between both taxa within their microhabitat, supported by the negative correlation (Supplementary Fig. S5).

Relative abundances of members of the fungal genus *Nectria* (OTU_1869, *Neonectria* sp. TB101 (*E*-value, 6e − 35, Ident, 81%)) were significantly reduced in HG and SG soils and were also negatively correlated to SDM and RDM. Such a negative correlation of *Nectria* sp. to apple plant growth was also reported for ARD soils^19^. Recent studies revealed that several fungal endophytes isolated from ARD-affected M26 roots were members of Nectriaceae, which in inoculation assays caused ARD symptoms^28^. Likewise, in infected rose fine roots, members of this fungal family were also detected by molecular tools^31^. Therefore, the detected sequences of *Nectria* in the RRD rhizosphere of the present study very likely contributed to the disease incidence of the *R. corymbifera* ‘Laxa’ plants. Members of *Cylindrocarpon*, also belonging to Nectriaceae, were repeatedly reported to cause ARD^23,26^. Likewise, the relative abundances of *Cylindrocarpon* sp. were reduced in this study in HG and SG soils (Table 2).

For the oomycete taxa, no common responders with significantly decreased relative abundance in response to the G treatment were identified (Table 4). A clear reduction was shown for *Peronospora* (OTU_2) with very high relative abundances in U (HU 53.10% and SU 23.97%) compared to G soils (both sites), but due to extremely high variability between single samples this difference was only significant for site H. This OTU also indicated a negative correlation to plant growth parameters (Supplementary Table S7). Members of *Peronospora* are well known to cause foliar diseases, i.e., downy mildew in sweet basil, onion, spinach, sugar beet, tobacco, opium poppy, and rose^47^. However, no studies have been reported so far that *Peronospora* sp. causes root diseases in plants. Thus, high relative abundance detected for members of *Peronospora* in RRD soil at both sites might be likely due to leaf residuals fallen onto the ground and to residing zoospores in the soils. To confirm this hypothesis, a more detailed investigation of longer and other *Peronospora* gene sequences potentially based on rhizosphere RNA rather than DNA is needed.

Some members of the oomycete genus *Globisporangium* were formerly classified as *Pythium*^48^. *Pythium* sp. have been listed as possible causal agents of ARD^3,23^. Thus, the members of *Globisporangium* (OTU_10 and OTU_279) that showed significantly reduced relative abundances in SG compared to SU soil (Table 4) might have contributed to the reduced plant growth in SU soil.

The relative abundance of the identified sequences assigned to *P. ramosum* was negatively correlated to the biomass of *R. corymbifera* ‘Laxa’ plants and it was reduced by γ-irradiation (Table 4 and Supplementary Fig. S7). Doan and Davis^49^ have recently reported that *Pythiogeton manoomin* isolated from infected roots and stems of wild rice was confirmed as a new species causing root and basal stalk rot of wild rice in the United States. The phylogenetic tree showed that this species was closely related to *P. ramosum*. Thus, the *Pythiogeton* members identified in the RRD soils of this study might have also contributed to RRD.

Quantification by quantitative PCR is recommended for future studies dealing with RRD to confirm the relative abundance of several of the identified responders, e.g., *Streptomyces*, *Nectria*, and *Pythiogeton*. Further, isolations followed by inoculation experiments to investigate their effects on plant performance and root morphology will lead to a better understanding of their role in the development of RRD. Studies using the same plant materials for rhizosphere microbial community and metabolite analyses should be considered for future investigations. Correlation analyses then can be applied to reveal relationships between root metabolites and microbial community composition in the respective rhizospheres.

## Conclusions

Our study revealed pronounced similarities between ARD symptoms on apple roots and RRD symptoms on rose roots at the microscopic level. In contrast to the highly increased levels of phytoalexins in apple roots affected by ARD, the phenolic secondary metabolites detected in rose roots from RRD soil did not show a clear trend. Only for aloesin, a significantly higher level was detected in roots grown in RRD soil from site H, suggesting this compound as a phytoalexin candidate of rose roots. Changes in the rhizosphere microbiome composition due to treatment effects correlated with changes in plant growth and root integrity and revealed Nectriaceae and *Streptomyces* as potential causal agents of RRD.

## Materials and methods

### Plant materials and growing conditions

Stratified seeds of *R. corymbifera* ‘Laxa’ were germinated in a growth chamber at 17 °C for 10 days and cultivated for another 18 days in the greenhouse where the experiment took place. The average temperature was 21 ± 1 °C. The relative humidity was adjusted to 70% and lowered to 50% 10 days after starting the experiment (on 20 February 2017). Additional light (SON-T Philips Master 400 W) was provided for 16 h per day in case the irradiation fell below 25 klux. The 4-week-old seedlings were about 10 cm in length (from root tip to shoot tip) and had two to three true leaves when used in the bioassay (Supplementary Fig. S1).

### Soils and soil treatments

Soils from two sites, Heidgraben (H) in Northern Germany (close to Hamburg) and Sangerhausen (S) from the European Rosarium, were collected. The soil H was taken at a depth of 0–20 cm, was of sandy texture with a pH of 5.4 (Supplementary Table S1) and was characterized previously by Mahnkopp et al.^1^. Starting in 2009, *R.* *corymbifera* ‘Laxa’ had been replanted at this site every second year. In contrast, soil from site S was loamy and had a pH of 7.0. At site S, the soil was exchanged after a certain time of cultivating roses in the exhibition area of the Rosarium, and this excavated soil (up to a depth of 1 m) was used in the experiment. Both soils were sieved (≤8 mm) and half of each soil was sent for γ-irradiation (at a minimal dose of 10 kGy; Beta-Gamma-Service, Wiehl, Germany). All four soil variants, e.g., untreated (U) and γ-irradiated (G) soils of both sites, were supplemented before potting with 2 g L^−1^ of the slow-release fertilizer Osmocote Exact 3-4 M (16-9-12 + 2MgO + trace elements; Everris International B.V., Geldermalsen, The Netherlands, https://icl-sf.com).

### Experimental design and measurement of plant growth parameters

Twenty 1 L pots were filled per soil variant and one seedling was planted per pot. The length of the main (longest) shoot was recorded weekly. Five each of the 20 plants per variant were destined for the microscopic investigations and the secondary metabolite analyses (LC-MS). After eight weeks, the final evaluation took place to record fresh and dry mass of the shoots and root fresh mass from ten plants per treatment. The roots were carefully washed, blotted dry and fresh masses were determined. Rhizospheres were extracted from five of the ten plants per variant, whereas RDM data (after drying for five days at 70 °C) were collected from the remaining five plants which were then used for metabolite analysis by GC-MS.

### Microscopy

Fine root samples were prepared as fresh whole-mounts for brightfield-microscopy with differential interference contrast to examine symptoms of RRD. Root segments from four plants (after cultivation for eight weeks) per treatment were analyzed, each with 30 random fresh root segments. For the detection of Actinobacteria by means of epi-fluorescence, the fluorochrome FUN 1^®^ Cell Stain (Thermo Fisher Scientific, Waldham, USA) was applied. Further root material of the plants from HU and HG soil was used for the histological examination of toluidine blue-stained thin sections^31^ to detect RRD-associated microorganisms in diseased tissues. All methods were used according to refs. ^2,31^.

### Analysis of secondary metabolites

For HPLC-HR-MS, roots were freeze-dried and 100 mg were extracted three times with 5 mL MeOH/DCM (7:1) by ultra-sonication for 5 min. The supernatants were combined. After evaporation of the solvent, the residue was reconstituted with 2 mL MeOH and centrifuged until clear supernatant could be collected. The samples were quantified alongside ^13^C-catechin as internal standard, using reversed-phase HPLC-HR-MS (Agilent 1260 LC-system and Orbitrap mass spectrometer; Thermo Fisher Scientific, Waltham, USA). Oven-dried roots were used for GC-MS analysis. Methodological details are provided as Supplementary Information 1.

### Characterization of the rhizosphere microbial communities

#### Collection of rhizosphere samples

Twenty rhizosphere samples (five per soil variant) were collected from roots of *R. corymbifera* ‘Laxa’ grown for eight weeks under greenhouse conditions in untreated (U) and γ-irradiated (G) RRD soils, from the two sites H and S. Soil was removed from plant roots through vigorous shaking. Subsequently, roots were cut into pieces of about 1 cm in length, which were carefully mixed. Depending on root biomass per plant (3–5 g), sterile 0.3% NaCl was added to the root segments in a stomacher bag. The roots were treated by a Stomacher 400 Circulator (Seward Ltd, Worthing, UK) for 30 s. The treatments were repeated three times to obtain a 1 : 10 dilution (w/v) and the supernatants were combined. The rhizosphere pellet was obtained by centrifugation at 5,000 *×* *g* for 30 min at 4 °C ^50^ and stored at −20 °C until total community (TC) DNA extraction.

#### Total community (TC-) DNA extraction and purification

Isolation of TC-DNA and purification from rhizosphere pellets was carried out using the FastDNA^®^ SPIN Kit and GENECLEAN^®^ SPIN Kit for soil (Qbiogene), respectively, according to the manufacturer’s guidance (MP Biomedicals, Heidelberg, Germany). Briefly, a direct extraction of the TC-DNA from the rhizosphere pellet sample was accomplished by bead beating for 60 s in the FastPrep^®^ Instrument from mpbio (MP Biomedicals, Santa Ana, CA, USA). Finally, the TC-DNA was eluted in 50 µl GENECLEAN^®^ SPIN elution solution^17^.

#### Library preparations of amplicons for bacteria, fungi, and oomycetes

Sequencing libraries for bacteria were prepared using a dual-PCR setup, targeting variable regions V3 and V4 of the 16S rRNA gene, approx. 460 bp^17^. In the first step, the primers Uni341F (5′-CCTAYGGGRBGCASCAG-3′) and Uni806R (5′-GGACTACNNGGGTATCTAAT-3′) were used^51^. Similarly, for ITS regions for fungi, the primers gITS7 (5′-GTGARTCATCGARTCTTTG-3′) and ITS4 (5′-TCCTCCGCTTATTGATATGC-3′) were applied to obtain fragments of about 211 bp of the ITS2 region^52^. In a second PCR step, the primers additionally included sequencing adapters and individual sample tags were used.

After both PCR reactions, amplicon products were purified using a HighPrep™ PCR Clean Up System (AC-60500, MagBio Genomics Inc., USA) applying a 0.65:1 (beads:PCR reaction) volumetric ratio to remove DNA fragments below 100 bp in size. Samples were normalized by SequalPrep Normalization Plate (96) Kit (Invitrogen, USA) and pooled using 5 µl volume each. The pooled sample libraries were concentrated using the DNA Clean and Concentrator™-5 kit (Zymo Research, Irvine, USA). The pooled library concentration was determined via the Quant-iT™ High-Sensitivity DNA Assay Kit (Life Technologies) and adjusted to 4 nM. Amplicon sequencing was performed on an Illumina MiSeq platform using Reagent Kit v2 [2 × 250 cycles] (Illumina, Inc., USA).

Rarefaction of 16S rRNA gene fragment sequence reads at 5,602 led to exclusion of two samples (HU14, 2313 reads and HG27, 5536 reads) from analyses, due to their lower read numbers. For the ITS sequences, samples SG26 (1896 reads), HU14 (7,151 reads), and HG13 (546 reads) were excluded from subsequent analyses after rarefying sequence reads at 10,554.

For oomycetes, the metabarcoding of a region of the mitochondrial cytochrome oxidase (cox-) subunit 2 was done^53^. In the first PCR, primers attached to Illumina overhang adapter sequences were used (Nex_Cox2hud-F: 5′-TCGTCGGCAGCGTCAGATGTGTATAAGAGACAGGGCAAATGGGTTTTCAAGATCC-3′ and Nex_cox2_233D8r: 5′-GTCTCGTGGGCTCGGAGATGTGTATAAGAGACAGGAATATTCATARS-TCCARTACC-3′). Amplified products were run on 1.5% agarose gels and bands of approximately 380 bp were excised and purified (NucleoSpin^®^ Gel and PCR Clean-up, Macherey-Nagel, Düren, Germany). Subsequently, index PCR of oomycetes was performed using Phusion High-Fidelity DNA polymerase (New England Biolabs, Frankfurt am Main, Germany) and products were purified by AMPure Beads (GENEWIZ, South Plainfield, USA)^54^. Each library was eluted in 25 µl nuclease free water and quantified using the Qubit dsDNA HS assay (Thermo Fisher Scientific, Waltham, USA). Subsequently, all samples were combined in equal concentrations to produce a 30 nM library. The pooled sample was further diluted to obtain a 20 nM library before sending for Illumina^®^ MiSeq^®^ paired-end sequencing^54^.

Two samples, namely SG3 (57 reads) and HU6 (341 reads) were excluded from subsequent analyses due to rarefaction of data at 775 reads.

#### Data analyses

To process 16S rRNA raw sequences, an in-house pipeline was followed. MiSeq Controller software was applied for sequence demultiplexing. BioDSL was employed to trim diversity spacers and sequencing adapters, to assemble sequence pair-reads, to remove short reads (<100 bp), to dereplicate remaining sequences and to get rid of singleton sequences (https://github.com/maasha/BioDSL). The GOLD database was employed to check and to remove chimera sequences^55^. Representative sequences were defined for each OTU with 80% confidence threshold based on bayesian estimates using Mothur v.1.25.0 default^56^. The reference OTU sequence was picked up by USEARCH v7.0.1090 using 97% similarity^57^. Representative sequences were classified against the Ribosomal Database Project (RDP) trainset9 database (032012)^58^. The sequence contingency table was exported on genus levels.

For ITS, an automated ITS pipeline, so-called PIPITS with default parameters for the ITS2 region was used to process ITS raw sequenced data according to Gweon et al.^59^. Briefly, the PIPITS_PREP generated raw reads from Illumina MiSeq sequencers for ITS files containing FASTA format and merged them into a single file for the next step. The PIPITS_FUNITS took this output as an input to identify the ITS subregion using HMMER3 according to Mistry et al.^60^. Then, clustering sequences into the OTU using VSEARCH (defined threshold at 97% sequence similarity), OTU abundance tables and the RDP taxonomic assignment table (using the UNITE fungal ITS reference data set) were generated by the PIPITS_PROCESS.

Regarding oomycetes, demultiplexed sequences and data analyses followed Sapp et al.^53^.

#### Statistical evaluation

The *t*-test was employed to test significant differences between the means (plant growth or secondary metabolites) of the two soil treatments (U and G). The PCoA based on Bray–Curtis dissimilarity metric, analysis of similarity (Anosim test), species richness and diversity indices namely Shannon^61^ and Chao1 diversity^62^ were evaluated on rarefied sequence data (at 5,602; 10,554 and 775 reads for bacteria; fungi and oomycetes; respectively). The PCoA and Anosim test were performed using the Past3 (3.02) software^63^. Normality of relative abundance distributions of rhizosphere bacteria and fungi Eq. (1) as well as oomycetes Eq. (2) was applied:

1 $${\it{sqrt}}\left( {\frac{n}{N} \ast 100 + 1} \right)$$

2 $${\mathrm{log}}_{{\mathrm{10}}}\left( {\frac{n}{N} \ast 100 + 1} \right)$$

where *n* was the numbers of sequences from each OTU and *N* was the total numbers of sequences in the sample. The Student’s *t*-test, function *t.test()* and a significant threshold of *p* *<* 0.05 was applied using the transformed data (eqs. (1) and (2), above) to check the effects of the soil treatments on the relative abundances of the rhizosphere microbiome with the software R-3.5.2. Any bacterial, fungal and oomycetes genera that presented significant differences in their relative abundances (>0.5%) between soil treatments were tested for correlation with SDM and root fresh mass of the plants using the Pearson’s correlation coefficient (*r*) by Past3 (3.02).

## Supplementary information

Supplemental Material

## Data Availability

All raw sequences (for bacteria, fungi, and oomycetes) were deposited at NCBI within the sequence read archive accession PRJNA627576. Further datasets analyzed during the current study are available in the BonaRes data repository, under the following doi numbers: 10.20387/bonares-1PDS-0X9R, 10.20387/bonares-HSQ7-8TBQ, 10.20387/bonares-KHE6-DEB9,10.20387/bonares-7XD5-STVN and 10.20387/bonares-B0F0-27SV.
