# Supplementary material for: Rhizosphere microbial communities associated to rose replant disease: links to plant growth and root metabolites
Source: Hortic Res. 2020 Sep 1;7:144. doi: 10.1038/s41438-020-00365-2 (PMC7459328; doi:10.1038/s41438-020-00365-2)
Supplement: Supplementary file 1 — Supplemental Material [file 41438_2020_365_MOESM1_ESM.docx]

# Rhizosphere microbial communities associated to rose replant disease: links to plant growth and root metabolites

B. Yim^1,2^, A. Baumann^1^, G. Grunewaldt-Stöcker^1^, B. Liu^3^, L. Beerhues^3^, S. Zühlke^4^, M. Sapp^5^, J. Nesme^6^, S.J. Sørensen^6^, K. Smalla^2^, and T. Winkelmann^1^

^1^ Institute of Horticultural Production Systems, Leibniz Universität Hannover, Hannover, Germany

^2^ Julius Kühn-Institut (JKI), Institute for Epidemiology and Pathogen Diagnostics, Braunschweig, Germany

^3^ Institute of Pharmaceutical Biology, Technische Universität Braunschweig, Braunschweig, Germany

^4^ Faculty of Chemistry and Chemical Biology (CCB), Technische Universität Dortmund, Dortmund, Germany

^5^ Cluster of Excellence on Plant Sciences (CEPLAS), Institute of Population Genetics, Heinrich Heine Universität, Düsseldorf, Germany

^6^ Section of Microbiology, Department of Biology, University of Copenhagen, Copenhagen, Denmark

**Supplementary information 1**

***Quantification of secondary metabolites via HPLC-HR-MS***

The chromatographic separation was carried out using a Luna® C18 (2) reversed phase column with a length of 50 mm, a diameter of 3 mm and a particle size of 3 μm (Phenomenex, Aschaffenburg, Germany). For the measurement, a gradient of H_2_O + 0.1 % TFA (trifluoroacetic) acid and acetonitrile + 0.1 % TFA with the following corner points was used. 0 minute: 100 % H_2_O and 0 % acetonitrile; 2 minutes: 100 % H_2_O and 0 % acetonitrile; 14 minutes: 0 % H_2_O and 100 % acetonitrile; 20 minutes: 0 % H_2_O and 100 % acetonitrile; 20.5 minutes: 100 % H_2_O and 0 % acetonitrile and 26 minutes: 100 % H_2_O and 0 % acetonitrile.

As ionization method, HESI II ion source (heated electrospray ionization) was used in positive mode. Detection was performed in full scan high resolution mode (resolving power 60000, mass deviation < 2 ppm, applying internal mass calibration). For quantification, the mixture of reference compounds contained 50, 100, 250, 500, 1000, 2500, 5000, 10000 and 20000 ng mL^-1^ of each compound to be quantified in MeOH, as well as 5000 ng ^13^C-catechin as ISTD (50 ng µL^-1^ in MeOH). For phlorizin, two additional concentrations (30000 ng mL^-1^ and 50000 ng mL^-1^ in MeOH HPLC-Grade) were added to the standard series. The data were evaluated using the software Xcalibur™ 2.1 by Thermo Fisher. The limit of detection (LOD) and the limit of quantification (LOQ) were set to signal to noise ratios of 3:1 and 9:1, respectively. For the quantification, root extracts were diluted 1:50 (1:2500 in case of phlorizin) using MeOH.

***Quantification of secondary metabolites via GC-MS***

For metabolite extraction and analysis were carried out as previously described by Weiß et al. (2017b)**^11^**. Briefly, 1 mL of methanol supplemented with 50 µg of 4-hydroxybiphenyl (internal standard for relative quantification) was added to the sample in a 2 mL reagent tube. The sample was continuously vortexed for 20 min at the maximum speed of 2,700 rpm. The resulting extract was centrifuged at 13,000 rpm for 10 min and the supernatant was transferred to a new tube. A 200 µL aliquot of the supernatant was transferred to a new 1.5 mL reagent tube. After drying under an air stream, the residue was re-dissolved in 200 µL ethyl acetate and centrifuged at 13,000 rpm for 10 min. The clear supernatant was transferred to a GC-MS vial with glass inlet. Ethyl acetate was evaporated by an air stream and the residue was silylated at 60 ℃ for 30 min using 50 µL N-methyl-N-(trimethylsilyl)trifluoroacetamide (MSTFA; ABCR, Karlsruhe, Germany). The silylated sample was analyzed by gas chromatography - mass spectrometry (GC-MS) using the following temperature program: 70 ℃ for 3 min, then linear increase of temperature from 70 ℃ - 310 ℃ over 24 min (10 ℃/min), and finally 310 ℃ for 5 min. Helium was the carrier gas with a flow rate of 1 mL/min. The injection volume was 1 μL with split ratio 1:10. Quantification of individual compounds was done based on the internal standard 4-phenylphenol. A response factor of 1 was assumed for all compounds.

**Supplementary tables**

**Table S1:** Soil texture distribution, pH and C and N contents

| **Site** | **Sand (%)** | **Silt (%)** | **Clay (%)** | **pH (CaCl_2)_** | **C (%)** | **N (%)** |
| --- | --- | --- | --- | --- | --- | --- |
| **H** | 93.2 | 3.8 | 3.0 | 5.42 | 2.6 | 0.2 |
| **S** | 38.5 | 44.5 | 17.0 | 6.95 | 2.9 | 0.2 |

H, Heidgraben and S, Sangerhausen

**Table S2:** Concentrations of plant available nutrients in soils from the two sites Heidgraben (H) and Sangerhausen (S), either untreated (U) or gamma-irradiated (G).

| **Nutrient** | **Concentration (mg g^-1^ soil) in different soil treatments** | | | |
| --- | --- | --- | --- | --- |
|  | **SU** | **SG** | **HU** | **HG** |
| **Si** | 0.62±0.002 | 0.59±0.005 | 0.03±0.004 | 0.04±0.003 |
| **Al** | 0.66±0.003 | 0.65±0.002 | 0.95±0.002 | 0.94±0.012 |
| **Ca** | 5.95±0.045 | 6.55±0.029 | 1.31±0.031 | 1.24±0.027 |
| **Cu** | 0.02±0.001 | 0.02±0.000 | 0.002±0.000 | 0.002±0.000 |
| **Fe** | 0.35±0.002 | 0.33±0.000 | 0.27±0.007 | 0.28±0.005 |
| **K** | 0.77±0.007 | 0.82±0.007 | 0.10±0.003 | 0.10±0.002 |
| **Mg** | 0.42±0.003 | 0.45±0.001 | 0.08±0.002 | 0.08±0.001 |
| **Mn** | 0.13±0.001 | 0.12±0.001 | 0.02±0.000 | 0.02±0.000 |
| **Na** | 0.003±0.003 | 0.001±0.000 | 0.000±0.000 | 0.000±0.000 |
| **P** | 0.37±0.002 | 0.42±0.001 | 0.340±0.002 | 0.334±0.005 |
| **Zn** | 0.07±0.001 | 0.08±0.000 | 0.013±0.000 | 0.012±0.000 |

Given are means±SD, measured by ICP-OES. n=3, except for Na from site H, n=2.

**Table S3:** Soil treatment and site effects on bacterial species richness and diversity indices in the rhizosphere of *R.* *corymbifera* ‘Laxa’ based on operational taxonomic units (OTUs) at 97% similarity

| **Site** | **Treatment** | **Sequences per condition** | **Number of  OTUs (97%)** | **Chao1** | **Shannon** |
| --- | --- | --- | --- | --- | --- |
| **H** | **HU** | 10329±797 | 351±42 | 427±41 | 4.43±0.31 |
|  | **HG** | 10485±5971 | 296±34 | 372±37 | 4.12±0.25 |
| **S** | **SU** | 8956±2716 | 391±26 a | 466±46 | 4.67±0.36 |
|  | **SG** | 10188±1736 | 343±22 b | 427±45 | 4.51±0.12 |

Mean±SD. Letters indicate significant differences between treatments within site using t-test, *p < 0.05,* and *n = 4* and *5* for sites H and S, respectively. Samples were taken at eight weeks after planting. U and G, untreated and gamma-irradiated RRD soil from sites Heidgraben (H) and Sangerhausen (S).

**Table S4:** Relative abundance (> 0.5%) of bacteria at genus level detected in the rhizosphere of *R.* *corymbifera* ‘Laxa’ grown for eight weeks in untreated (U) and gamma-irradiated (G) RRD soils from two sites (S and H).

| **Phylum** | **Genus** | **OTU_ID** | **Site H** | | **Site S** | |
| --- | --- | --- | --- | --- | --- | --- |
|  |  |  | **HU** | **HG** | **SU** | **SG** |
| **Acidobacteria** | ***Gp3*** | OTU_30 | 0.04±0.01 a | 0.00±0.01 b | 0.07±0.04 a | 0.73±0.37 b |
| **Actinobacteria** | ***Streptomyces*** | OTU_64 | 1.91±1.07 a | 0.23±0.13 b | 1.16±0.46 a | 0.32±0.09 b |
| **Bacteroidetes** | ***Ohtaekwangia*** | OTU_140 | 0.01±0.02 | 0.00±0.00 | 0.84±0.26 a | 0.01±0.01 b |
|  | ***Lacibacter*** | OTU_3 | 0.00±0.00 | 0.00±0.01 | 0.66±0.17 a | 3.67±1.69 b |
|  | ***Niastella*** | OTU_36 | 0.05±0.03 a | 0.00±0.00 b | 3.43±1.70 a | 0.04±0.03 b |
|  | ***Flavitalea*** | OTU_524 | 0.03±0.03 | 0.29±0.31 | 0.38±0.23 a | 0.74±0.16 b |
| **Chloroflexi** | ***Herpetosiphon*** | OTU_141 | 0.00±0.00 | 0.00±0.00 | 0.00±0.00 a | 0.57±0.33 b |
|  | ***Caldilinea*** | OTU_113 | 0.04±0.04 | 0.00±0.00 | 0.01±0.01 a | 0.54±0.15 b |
|  | ***Herpetosiphon*** | OTU_35 | 0.00±0.00 | 0.03±0.02 | 0.00±0.01 a | 2.08±1.20 b |
|  | ***Herpetosiphon*** | OTU_39 | 0.00±0.00 | 0.02±0.04 | 0.01±0.02 a | 1.77±1.29 b |
| **Proteobacteria** | ***Bordetella*** | OTU_1 | 0.03±0.01 a | 9.42±8.55 b | 0.01±0.01 | 0.00±0.00 |
|  | ***Microvirga*** | OTU_107 | 0.00±0.01 a | 0.37±0.26 b | 0.15±0.06 a | 1.16±0.29 b |
|  | ***Acidovorax*** | OTU_14 | 0.03±0.03 | 0.02±0.02 | 1.52±0.59 a | 0.47±0.27 b |
|  | ***Mesorhizobium*** | OTU_143 | 0.78±0.27 a | 0.17±0.06 b | 0.31±0.11 a | 0.55±0.13 b |
|  | ***Novosphingobium*** | OTU_17 | 5.64±1.45 a | 1.84±1.01 b | 1.72±0.50 | 0.93±0.75 |
|  | ***Methylophilus*** | OTU_24 | 0.02±0.02 | 0.01±0.01 | 0.30±0.12 a | 1.88±0.33 b |
|  | ***Sphingobium*** | OTU_33 | 0.57±0.41 a | 0.03±0.02 b | 1.06±0.77 | 0.74±0.72 |
|  | ***Sphingobium*** | OTU_7 | 2.78±3.14 a | 0.06±0.12 b | 0.79±0.33 a | 0.05±0.05 b |
|  | ***Sphingopyxis*** | OTU_339 | 1.30±0.59 a | 0.02±0.01 b | 0.27±0.11 a | 0.08±0.07 b |
|  | ***Acidovorax*** | OTU_423 | 0.19±0.15 a | 0.58±0.25 b | 0.90±0.52 | 0.70±0.60 |
|  | ***Acidovorax*** | OTU_474 | 0.19±0.13 | 0.06±0.04 | 0.51±0.14 a | 0.10±0.07 b |
|  | ***Asticcacaulis*** | OTU_424 | 1.06±0.32 a | 0.38±0.24 b | 0.06±0.02 | 0.14±0.07 |
|  | ***Hydrogenophaga*** | OTU_47 | 0.00±0.00 | 0.00±0.00 | 0.61±0.26 a | 0.14±0.13 b |
|  | ***Hydrogenophaga*** | OTU_50 | 0.01±0.01 | 0.01±0.03 | 0.21±0.07 a | 1.15±0.44 b |
|  | ***Vampirovibrio*** | OTU_52 | 0.00±0.00 | 0.00±0.00 | 1.31±0.83 a | 0.00±0.00 b |
|  | ***Rhizobacter*** | OTU_63 | 0.00±0.01 | 0.00±0.01 | 0.68±0.31 a | 0.04±0.07 b |
|  | ***Phenylobacterium*** | OTU_78 | 0.89±0.32 | 0.41±0.41 | 0.13±0.07 a | 0.02±0.02 b |
|  | ***Rhizobium*** | OTU_8 | 4.43±1.04 a | 0.14±0.08 b | 0.99±0.61 a | 0.11±0.08 b |
|  | ***Bradyrhizobium*** | OTU_877 | 0.66±0.30 | 0.89±0.47 | 0.38±0.07 a | 0.20±0.09 b |
| **TM7** | ***TM7_genus_incertae_sedis*** | OTU_42 | 0.57±0.37 a | 0.00±0.00 b | 1.35±0.58 a | 0.01±0.01 b |
|  | ***TM7_genus_incertae_sedis*** | OTU_9 | 0.61±0.18 | 1.04±1.01 | 0.20±0.15 a | 0.02±0.02 b |

Mean±SD. Letters indicate significant differences between treatments within site and genus, t-test, *p < 0.0*5 and *n = 4* and *5* for sites H and S, respectively. Colored cells indicate common responders found at both sites. Significantly decreased and increased bacterial relative abundance in G (gamma-irradiated) compared to U (untreated) soil is highlighted in red and green, respectively. OTU, operational taxonomic unit.

**Table S5:** Soil treatment and site effects on fungal species richness and diversity indices in the rhizosphere of *R.* *corymbifera* ‘Laxa’ based on operational taxonomic units (OTUs) at 97% similarity

| **Site** | **Treatment** | **Sequences per condition** | **Number of OTUs (97%)** | **Chao1** | **Shannon** |
| --- | --- | --- | --- | --- | --- |
| **H** | **HU** | 22333±9354 | 551±236 | 1082±440 | 2.90±1.32 |
|  | **HG** | 18358±10190 | 440±189 | 774±307 | 3.22±0.87 |
| **S** | **SU** | 22841±4999 | 564±131 a | 1062±191 a | 2.09±0.75 |
|  | **SG** | 28502±5164 | 193±98 b | 397±145 b | 1.11±1.07 |

Mean±SD. Letters indicate significant differences between treatments within site using t-test, *p < 0.05* and *n = 4*, except for SU with *n = 5*. Samples were taken at eight weeks after planting. U and G, untreated and gamma-irradiated RRD soil, respectively.

**Table S6:** Soil treatments and site effects on oomycetes species richness and diversity indices in the rhizosphere of *R.* *corymbifera* ‘Laxa’ based on operational taxonomic units (OTUs) at 97% similarity

| **Site** | **Treatment** | **Sequences per condition** | **Number of OTUs (97%)** | **Chao1** | **Shannon** |
| --- | --- | --- | --- | --- | --- |
| **H** | **HU** | 25505±34817 | 8±0 | 8±0 | 0.95±0.16 |
|  | **HG** | 23379±21490 | 7±2 | 7±2 | 0.91±0.64 |
| **S** | **SU** | 1755±728 | 12±3 | 14±2 | 1.22±0.62 |
|  | **SG** | 3939±4037 | 6±4 | 6±4 | 0.50±0.50 |

Mean±SD. No significant differences detected between treatments within site using t-test, *p < 0.05* and n = 4 for HU and SG and n = 5 for HG and SU. Samples were taken at eight weeks after planting. U and G, untreated and gamma-irradiated RRD soil, respectively.

**Table S7:** Pearson correlation between relative abundances (> 0.5%) of cox2 gene fragments detected in rhizosphere and shoot dry mass (SDM) and root fresh mass (RFM) of *R.* *corymbifera* ‘Laxa’ eight weeks after planting

| **Species** | **OTU_ID** | **Relative  abundance** | **SDM** | | **RFM** | |
| --- | --- | --- | --- | --- | --- | --- |
|  |  |  | ***r value*** | ***p value*** | ***r value*** | ***p value*** |
| ***Peronospora potentillae-anserinae*** | **OTU_2** | 18.75±32.73 | -0.35 | 0.15 | -0.48 | 0.04 |
| ***Pythiogeton ramosum*** | **OTU_349** | 1.10±1.87 | -0.39 | 0.11 | -0.47 | 0.04 |

Mean±SD, relative abundance (n = 18), OTU, operational taxonomic unit. Negative significant correlation to SDM or RFM of *R. corymbifera* ‘Laxa’ plants is highlighted in red. Data present only those taxa that showed significant correlation to SDM or RFM.

**Supplementary figures**


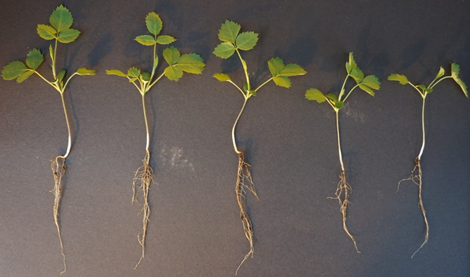


**Figure S1:** Plant materials for the greenhouse bioassay: four-week-old seedlings of *R. corymbifera* ‘Laxa‘. Bar represents 1 cm.

**
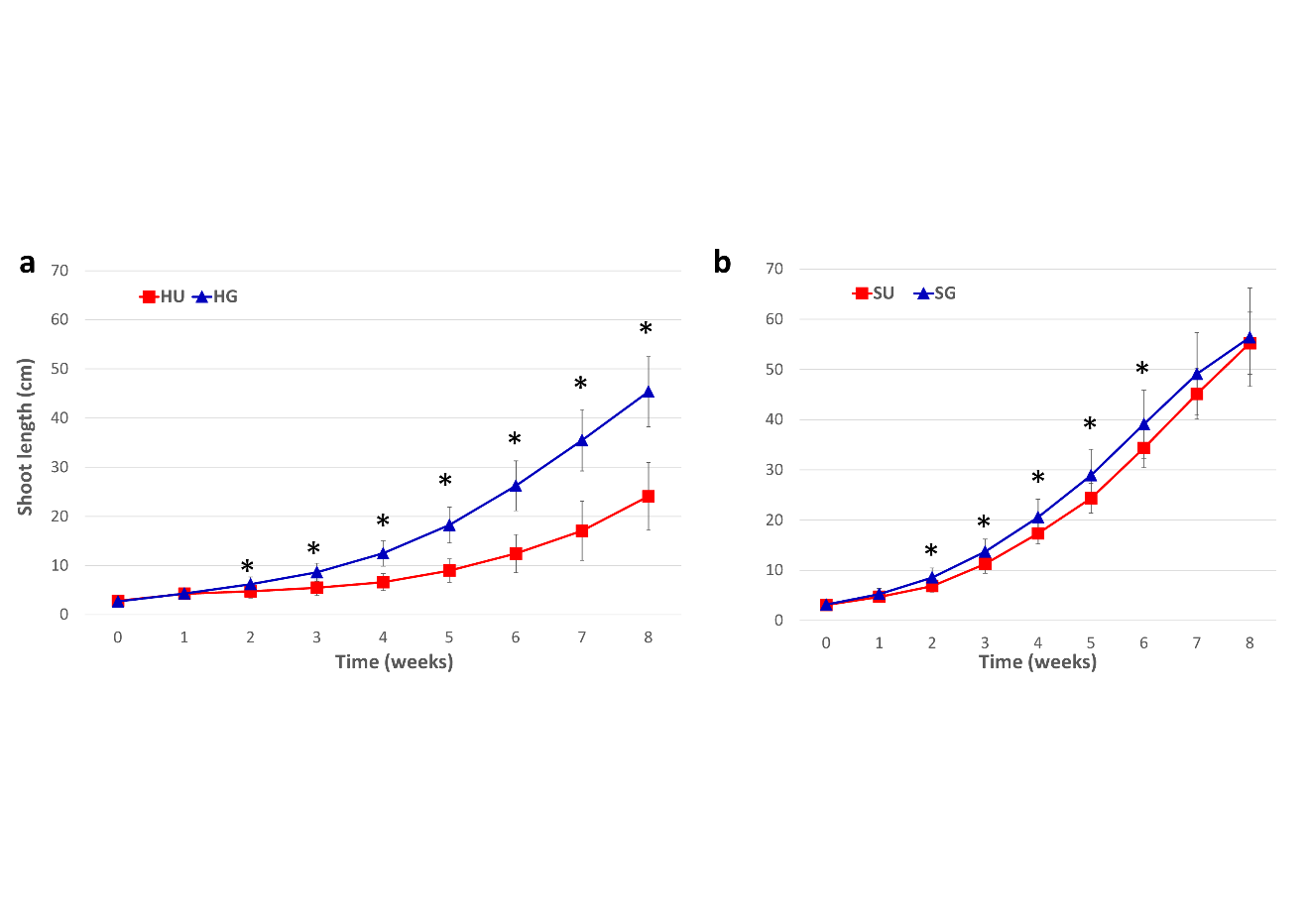
**

**Figure S2:** Shoot growth of *R.* *corymbifera* ‘Laxa’ under greenhouse conditions (bioassay) in soil from Heidgraben (A) and Sangerhausen (B). Indicated are means and standard deviations of 10 plants per variant. Asterisks indicate significant differences between untreated (U) and gamma-irradiated (G) variants within site (t-test and *p < 0.05*).


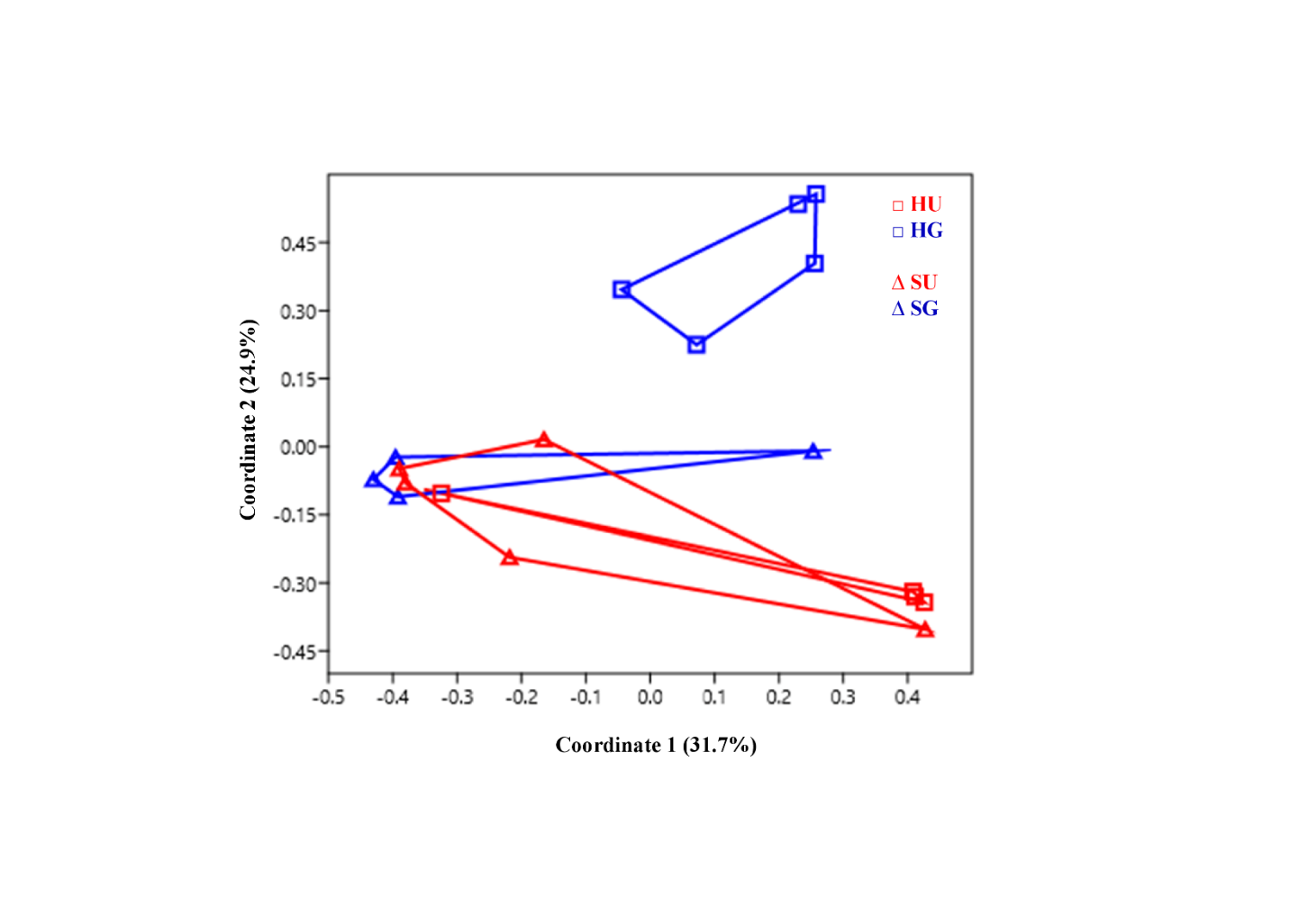
**Figure S3:** Oomycete community composition in the rhizosphere of *R.* *corymbifera* ‘Laxa’ grown for eight weeks in untreated (U) and gamma-irradiated (G) RRD soils from two sites (S and H) eight weeks after planting, revealed by principal coordinate analysis (PCoA) using Bray-Curtis distance metric, based on operational taxonomic units (OTUs). Past3, n = 4 for HU and SG and n = 5 for HG and SU.

**
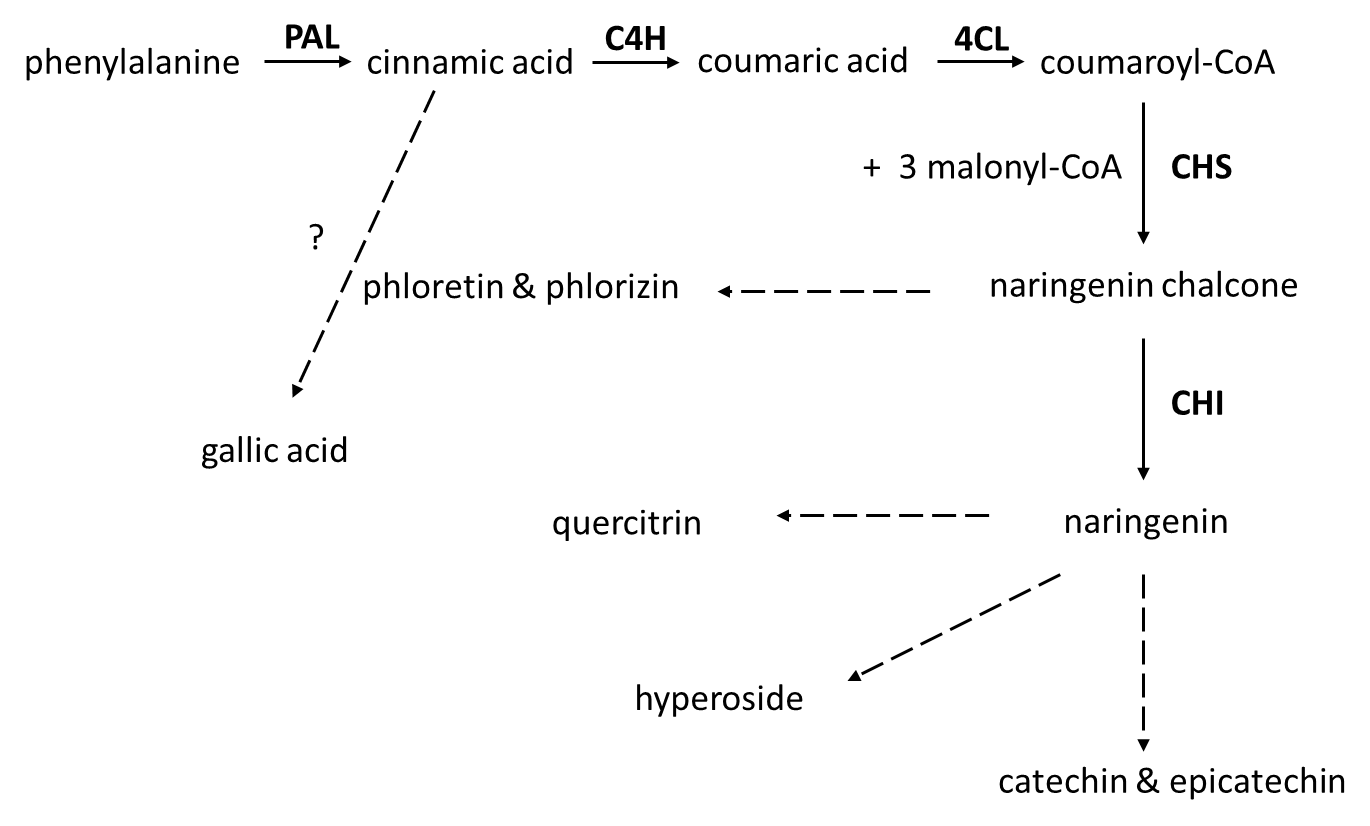
**

**Figure S4:** Schematic illustration of flavonoid biosynthesis. Solid arrows indicate one step reactions and the respective enzymes (PAL = phenylalanine ammonia-lyase, C4H = cinnamate 4-hydroxylase, 4CL = 4-coumaric acid:coenzyme A ligase, CHS = chalcone synthase, CHI = chalcone isomerase), whereas broken arrows indicate multiple-step reactions. Arrows with question mark: unconfirmed steps.


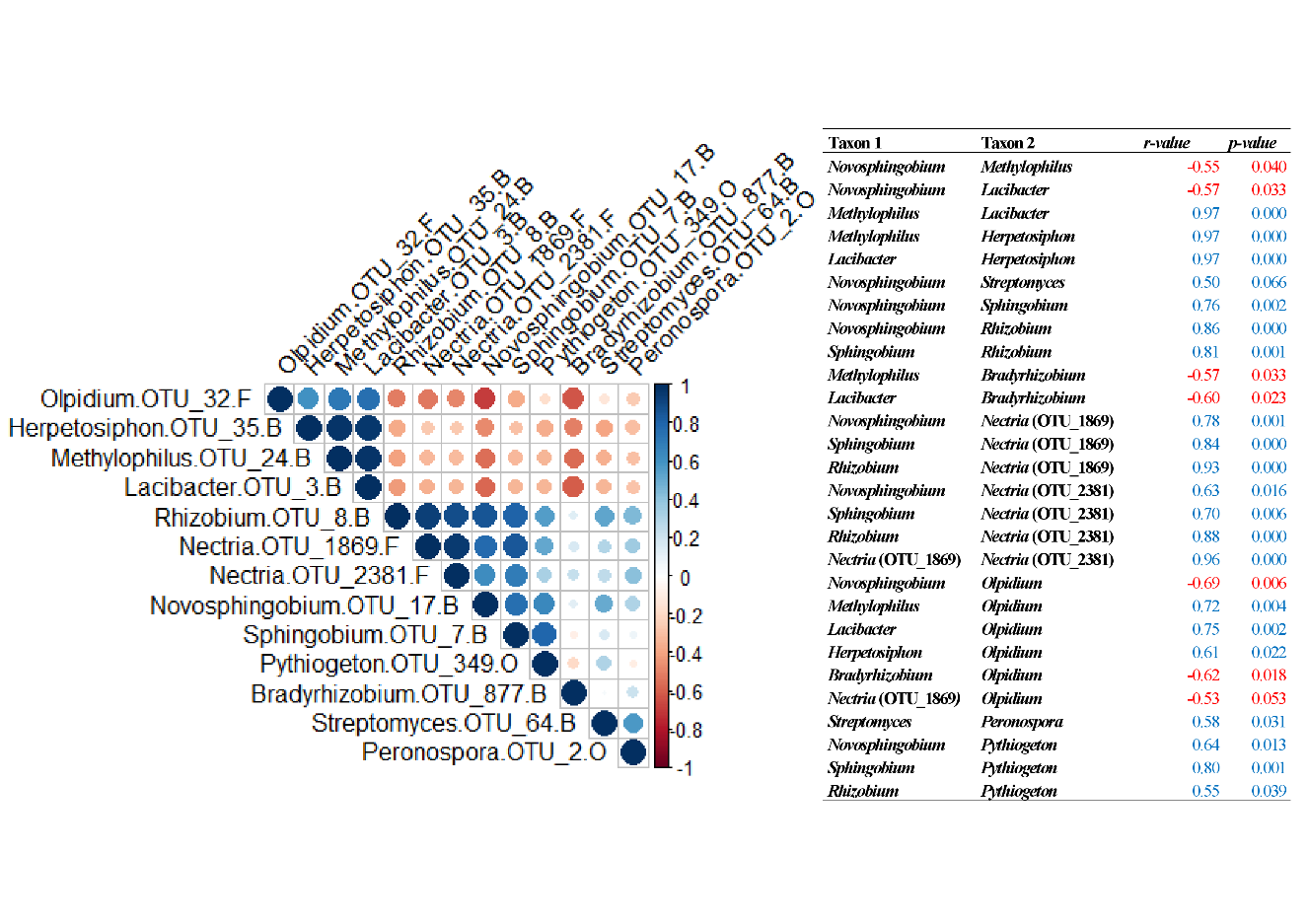


**Figure S5:** Spearman correlation coefficient between relative abundances of bacterial (OTU_ID.B), fungal (OTU_ID.F) and oomycetes (OTU_ID.O) communities in the rhizosphere of *R. corymbifera* ‘Laxa’ after growing for eight weeks under greenhouse conditions. The Spearman correlation was applied only to those taxa, whose relative abundances (> 0.5%) significantly correlated to either shoot or root dry mass of the plants. The pairwise correlation values (r- and p-values) are presented only for those taxa that showed significant positive (in blue color) or negative (in red color) correlation.
